# Cage Balancing Enhances Optoelectronic and Lasing Performance in Stable Quasi-2D Tin Iodide Perovskites

**DOI:** 10.1021/jacs.5c09938

**Published:** 2025-09-15

**Authors:** Christopher T. Triggs, Chun-Sheng Jack Wu, Yarong He, Eliana Bernat, Willa Mihalyi-Koch, Kristel M. Forlano, Ilia A. Guzei, Daniele Cortecchia, Annamaria Petrozza, Song Jin

**Affiliations:** † Department of Chemistry, 5228 University of Wisconsin-Madison, Madison, Wisconsin 53703, United States; ‡ Center for Nanoscience and Technology, 9296 Istituto Italiano di Tecnologia, via Rubattino 81, Milano 20134, Italy; § Department of Industrial Chemistry Toso Montanari, University of Bologna, via Piero Gobetti 85, Bologna 40129, Italy

## Abstract

Two-dimensional (2D) tin halide perovskites are highly tunable and low-toxicity semiconductors, promising for next-generation optoelectronics. However, achieving air stability and excellent photophysical properties simultaneously necessitates deliberate structure tuning using organic spacer cations and A-site cations. Here, we report a series of new quasi-2D Ruddlesden–Popper tin halide perovskites using a fluorinated aromatic spacer cation, 4-fluorophenethylammonium (4FPEA), and systematically investigate the impacts of layer thickness, spacer cation, and A-site cation on the crystal structures and optical properties of (4FPEA)_2_(A)_*n*−1_Sn_*n*_I_3*n*+1_. These 4FPEA-based 2D tin perovskites, further tuned by the A-cations, exhibit uniquely undistorted 180° out-of-plane Sn–I–Sn bond angles and low octahedral distortions compared to other quasi-2D perovskites and demonstrate prolonged air stability, excellent photophysics, and amplified spontaneous emission and lasing in exfoliated microflakes. A comprehensive survey of reported *n* = 2 lead and tin iodide perovskites reveals that all structures can be classified into three types (tilted, balanced, and buckled) based on the structural distortion parameters of their perovskite cages. Notably, (4FPEA)_2_(A)­Sn_2_I_7_ are among the handful of “balanced” *n* = 2 perovskites with minimal distortion and excellent optoelectronic performance. The structural insights and cage-balancing approach revealed herein motivate the deliberate design of quasi-2D perovskites through the synergy of the spacer and cage cations, further paving the way for high-performance optoelectronic applications of stable tin halide perovskites.

## Graphical abstract

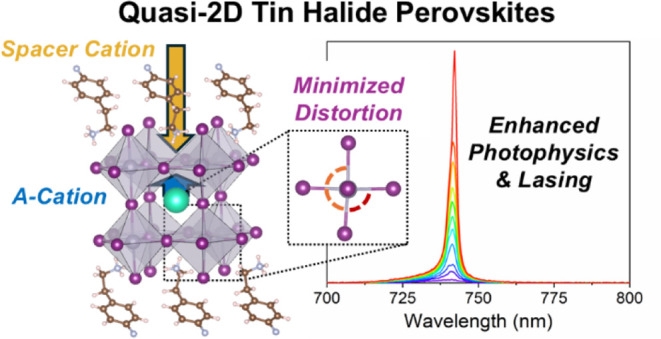

## Introduction

Two-dimensional (2D) Ruddlesden–Popper (RP) metal halide perovskites are versatile next-generation semiconductors^1,2^ with high-performance applications in solar cells,^3–6^ light emitting diodes,^7–9^ field effect transistors,^10–12^ and beyond.^13,14^ Such versatility arises from their tunable multicomponent crystal structure with the general formula (LA)_2_(A)_*n*−1_B_*n*_X_3*n*+1_, where B is a divalent metal (generally Pb^2+^ or Sn^2+^), X is a halide anion, A is a small monovalent cation (Cs^+^, methylammonium (MA), or formamidinium (FA)), and LA is a large organic spacer cation that truncates the inorganic network into 2D sheets of *n* thickness.^2,15,16^ Compared to the extensively studied lead-based Ruddlesden–Popper perovskites,^2^ nontoxic tin-based 2D perovskites have only recently garnered much attention for their greater environmental and biological safety,^17^ narrower bandgaps in the red and NIR regime,^18–20^ robust exciton dynamics,^20–22^ high carrier mobilities,^10,16^ high quantum efficiencies and high color purity.^9,23,24^ Owing to these excellent photophysical properties, amplified spontaneous emission and lasing have been readily attained in 2D (*n* = 1) and quasi-2D (*n* > 1) tin iodide perovskites,^25,26^ which has been generally difficult to achieve in comparable 2D lead iodide perovskites without high layer numbers (*n* ≥ 3),^27,28^ elaborate semiconducting spacer cations,^26,28^ or engineered nanowire morphologies that can serve as waveguides.^29,30^ These suggest tin-based perovskites are highly promising materials for high-performance optoelectronics. However, the sensitivity of Sn^2+^ to oxygen and moisture results in gradual oxidation and loss of properties, inhibiting the practical applications of these lead-free materials.^31–33^ On this note, the air sensitive inorganic SnX_6_ networks can be better protected by bulkier organic spacer cations that are halogenated or divalent enabling tighter interlayer packing and providing enhanced stability of 2D tin perovskites.^34–36^ Careful design of tin halide perovskite crystal structures by deliberate selection of organic cations is required to maximize optoelectronic performance and air stability simultaneously in order to take full advantage of their properties.

At the forefront of high-performance RP perovskites are quasi-2D (*n* > 1) phases that exhibit further decreased bandgaps, longer carrier lifetimes, and lower exciton binding energies compared to the *n =* 1 prototypes.^7,37–39^ Structurally, quasi-2D perovskites possess multilayered corner-sharing BX_6_ network structures that are still confined by large spacer cations but also incorporate small A-site cations in perovskite cages (i.e., the cuboid unit formed by eight neighboring BX_6_ octahedra, Figure 1),^15^ thereby allowing for more complex structural chemistry and tunability that cannot be obtained in 2D (*n* = 1 monolayered structures) or 3D (*n* = ∞) analogues.^15,19,40,41^ Even though the organic spacer and A-cations generally do not directly contribute to the electronic structures of quasi-2D perovskites, the structure features modulated by organic cations, such as B–X bond angles/lengths, octahedral distortions, B–X orbital overlap, can have significant impacts on the photophysical characteristics (bandgaps, electron–phonon interactions, carrier relaxation, etc.), and ultimately the optoelectronic performance (photoluminescence lifetimes and quantum yields, etc.).^2,42–48^ Lead-based quasi-2D perovskites have been studied more extensively with a variety of spacer cations and A-cations.^2,40,41^ In contrast, reports of *n* > 1 tin halide perovskites have been more limited,^12,19–22,26,36,49–51^ and have generally focused on the common butylammonium (BA) and phenethylammonium (PEA) spacer cations that have limited protective capabilities for the air sensitive tin perovskites.^34–36,52^ The ideal spacer cation for quasi-2D tin perovskites should enable high air stability and enhanced optoelectronic performance simultaneously. However, design insights for quasi-2D perovskites have been generally lacking due to their complicated multicomponent structures (Figure 1) that are synergistically tuned by the complex interplay between the spacer and A-cations.^46^ A clear description of the structural distortions in quasi-2D perovskites and a broad understanding of how such structural distortions are influenced by the organic cations is required to develop such insights.

**Figure 1:**
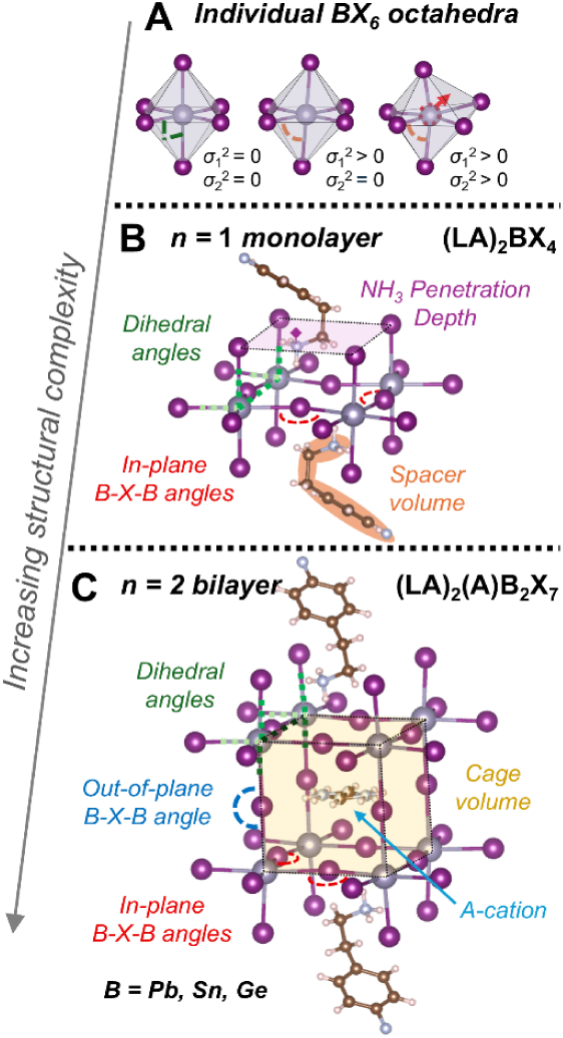
Schematic structures depicting the increasing structural complexity in 2D halide perovskites. Starting from the building blocks of individual BX_6_ octahedra, the possible structural distortions and the number of structural parameters become increasingly complex with increasing layer thickness. In the *n* = 2 (quasi-2D) perovskite case, both the spacer cation (LA) and A-cation modulate the perovskite cage and the various structural parameters and distortions.

2D halide perovskites are constructed from corner-sharing BX_6_ octahedra, whose structural distortions are quantified by the bond angle variance parameters σ_1_^2^ and σ_2_^2^ that describe deviations from the ideal 90° and 180° metal-halide bond angles within an octahedron (where α_*i*_ and β_*i*_ are neighboring and opposite of X–B–X bond angles, respectively).^53–55^ Similarly, the bond distortion index *D* describes the deviation of individual B–X bonds *d*_*i*_ from the average B–X bond distance.

$$\sigma_{1}^{2}=\frac{1}{11}\overset{12}{\underset{i=1}{\sum }}{\left(\alpha_{i}-90\right)}^{2}$$

$$\sigma_{2}^{2}=\frac{1}{2}\overset{3}{\underset{i=1}{\sum }}{\left(\beta_{i}-180\right)}^{2}$$

$$D=\frac{1}{6}\overset{6}{\underset{i=1}{\sum }}\left|\frac{\left(d_{i}-d_{ave}\right)}{d_{ave}}\right|$$

The ideal undistorted perovskite octahedron exhibits σ^2^ and *D* values of zero but most octahedra exhibit some distortions (i.e., nonzero σ^2^ and *D* values) (Figure 1A). Building upon these intraoctahedral distortions, the assembly of these octahedra into *n* = 1 monolayers produces variations in how the octahedra are tilted relative to one another (i.e., interoctahedral distortions), which can be described by the in-plane B–X–B angles and dihedral angles (Figure 1B). In the well-studied *n* = 1 (LA)_2_BX_4_ perovskites, these structural distortions are dictated by the strain imparted by the spacer cations via ammonium headgroup size and penetration and interlayer interactions, dependent on the metal and halide identity, and are intimately tied to their photophysical performance.^42,44,55–60^ However, such correlations are unclear beyond *n* = 1, as the buildup from monolayer networks to bilayer networks of BX_6_ octahedra (*n* = 2) introduces additional complexity in how the layers of octahedra are stacked onto one another (i.e., the out-of-plane B–X–B bond angle) to form the perovskite cages that house the A-cations (Figure 1C). The inequivalent strains imparted by the A-cation and spacer cation from the inside and outside of the perovskite cage often cause nonzero values of σ_1_^2^ and σ_2_^2^.^19,20,40,54^ Therefore, in quasi-2D *n* = 2 (LA)_2_(A)­B_2_X_7_ perovskites, the intraoctahedral distortions (σ_1_^2^ and σ_2_^2^), interoctahedral distortions (various B–I–B angles and dihedral angles), and cage volume and geometry are all modulated by the complex interplay between the spacer and A-site cations. Systematic study of quasi-2D tin perovskite single crystal structures is necessary to develop design insights for these structurally complex but highly promising hybrid materials.

In this work, we report a series of four new quasi-2D tin halide perovskites based on the 4-fluorophenethylammonium (4FPEA) spacer cation, (4FPEA)_2_(A)_*n*−1_Sn_*n*_I_3*n*+1_ (*n* = 2 and *n* = 3), and comprehensively analyze the impacts of dimensionality, spacer cations, and A-cations to better understand their fundamental structure–property relationships. Fluorinated PEA is known to enable stronger molecular dipoles and dielectric confinement, greater hydrophobicity, and stability compared to PEA in lead based perovskites,^61–63^ but so far 4FPEA has had limited study in tin perovskites except in the 2D monolayer case (*n* = 1),^64,65^ or as additives in thin film photovoltaics that provide greater crystallinity and stability.^66–68^ The new (4FPEA)_2_(A)­Sn_2_I_7_ (A = Cs^+^, MA, FA) structures exhibit uniquely undistorted out-of-plane Sn–I–Sn bond angles (i.e., near or at 180°), in addition to minimized dihedral angles and octahedral distortions, compared to other *n* = 2 (LA)_2_(A)­Sn_2_I_7_ tin perovskites with various spacer cations. Notably, (4FPEA)_2_(Cs)­Sn_2_I_7_ is a rare example of an *n* = 2 lead or tin iodide perovskite crystal structure that incorporates the smaller Cs^+^ cation. The minimal structural distortion, excellent air-stability, and excellent photophysical properties in (4FPEA)_2_(A)_*n*−1_Sn_*n*_I_3*n*+1_ enable low-temperature amplified spontaneous emission (ASE), and sometimes whispering gallery mode lasing, in microflakes of each of the 4FPEA)_2_(A)_*n*−1_Sn_*n*_I_3*n*+1_ structures. Furthermore, through a comprehensive survey, we found that quasi-2D lead and tin iodide perovskites can be sorted into “tilted”, “balanced”, or “buckled” cage types based on structural parameters that describe the different distortions present in the perovskite cages, and 4FPEA-based structures are among the few with balanced perovskite cages. The structural insights revealed herein underscore how the spacer cation and cage cations can synergistically enhance stability and balance the quasi-2D perovskite cage for improved optoelectronic properties.

## Results and Discussion

### Layer Thickness Effects in (4FPEA)_2_(A)_*n*−1_Sn_*n*_I_3*n*+1_ (*n* = 1–3)

We synthesized 4FPEA-based quasi-2D tin perovskites via the conventional slow cooling crystallization method in hydroiodic acid using off-stoichiometry recipes (see Experimental Details and Table S1 in the Supporting Information). Careful optimization of the precursor stoichiometries yielded large and phase-pure single crystals of *n* = 2 (4FPEA)_2_(A)­Sn_2_I_7_ (A = Cs^+^, MA, FA) and *n* = 3 (4FPEA)_2_(MA)_2_Sn_3_I_10_ perovskites that were studied using single-crystal X-ray diffraction to determine their crystal structures (Figure 2A–C and Table 1). The stoichiometry optimization was particularly important for (4FPEA)_2_(Cs)­Sn_2_I_7_ and demanded the addition of low stoichiometric ratios of CsI at high temperature to prevent cocrystallization of undesired byproducts. For the *n* = 3 structure, an aqueous/organic solvent mixture was required to obtain large and phase-pure single crystals. We note that *n* = 3 tin halide perovskites are even less common in the literature, with previously reported crystal structures only based on the BA and PEA spacer cations.^12,21^

**Figure 2:**
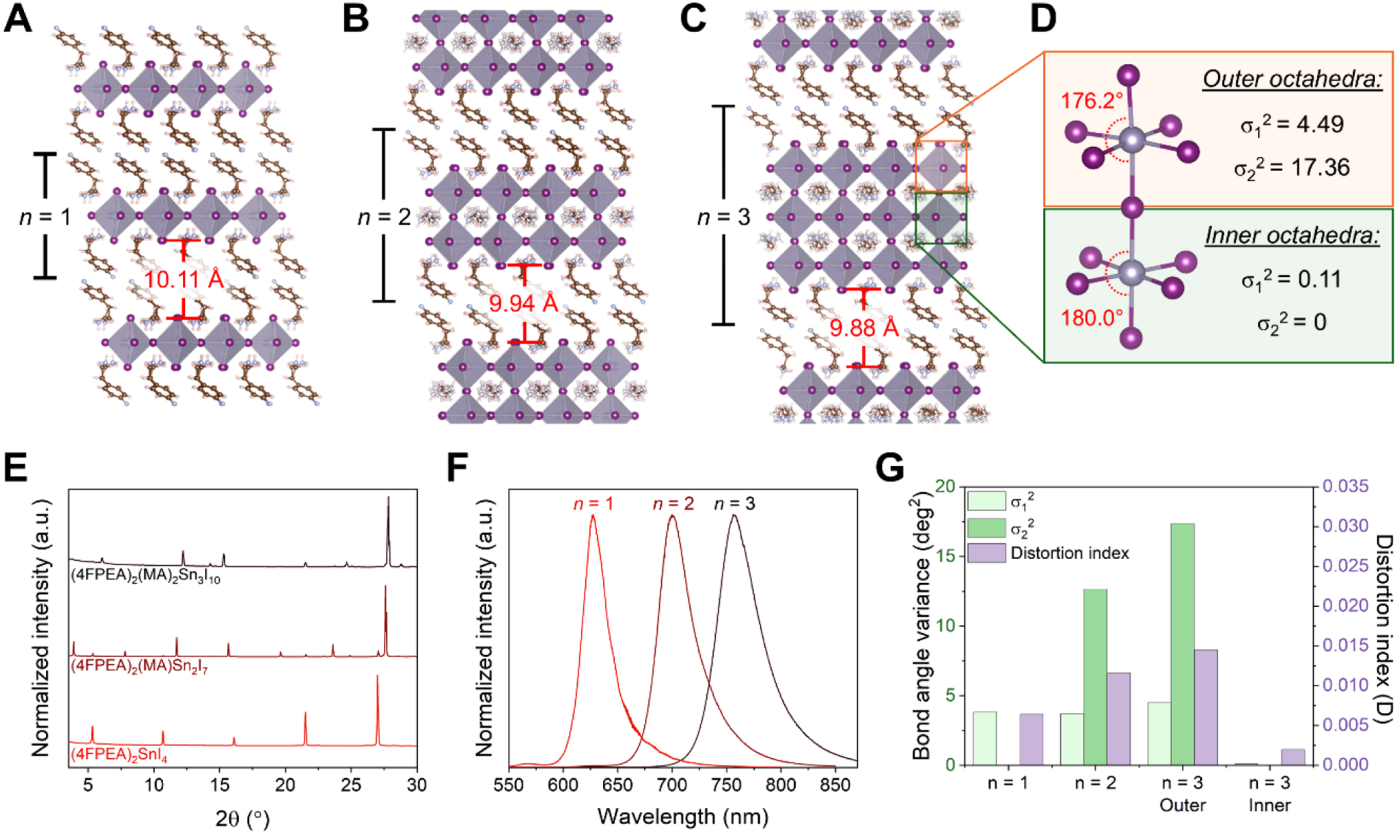
Dimensionality effects on the structures and properties of (4FPEA)_2_(MA)_*n*−1_Sn_*n*_I_3*n*+1_ (*n* = 1–3) perovskites. Room-temperature crystal structures for (A) *n* = 1 (4FPEA)_2_SnI_4_ [CIF adapted from ref 64. Copyright 2001 American Chemical Society], (B) *n* = 2 (4FPEA)_2_(MA)­Sn_2_I_7_, and (C) *n* = 3 (4FPEA)_2_(MA)_2_Sn_3_I_10_ with (D) a perspective comparing the inner versus outer SnI_6_ octahedra. The corresponding (E) powder X-ray diffraction patterns, (F) PL spectra, and (G) calculated bond angle variances (σ_1_^2^ and σ_2_^2^) and bond distortion index (*D*) for (4FPEA)_2_(MA)_*n*−1_Sn_*n*_I_3*n*+1_ perovskites (*n* = 1–3).

**Table 1:** Crystallographic and Structure Refinement Data for the New 4FPEA-Based Quasi-2D Tin Halide Perovskite Reported Herein (295 K)

Compound	(4FPEA)_2_(Cs)Sn_2_I_7_	(4FPEA)_2_(MA)Sn_2_I_7_	(4FPEA)_2_(FA)Sn_2_I_7_	(4FPEA)_2_(MA)_2_Sn_3_I_10_
Crystal System	monoclinic	monoclinic	monoclinic	monoclinic
Space group	*P*2_1_/*c*	*C*2/*c*	*P*2_1_/*c*	*P*2_1_/*c*
*a*/Å	22.701(9)	45.350(9)	22.636(4)	28.8881(18)
*b*/Å	8.615(4)	8.6744(17)	8.7406(14)	8.7088(6)
*c*/Å	8.732(5)	8.7796(18)	8.8426(15)	8.7901(5)
β/deg	96.13(4)	95.05(3)	96.044(2)	94.759(4)
Volume/Å^3^	1697.9(14)	3440.3(12)	1739.8(5)	2203.8(2)
*Z*	2	4	2	2
ρ_calc_, g/cm^3^	3.010	2.776	2.770	2.968
Final *R* indexes [I ≥ 2σ (I)]	*R*_1_ = 0.0451, *wR*_2_ = 0.1005	*R*_1_ = 0.0464, *wR*_2_ = 0.0761	*R*_1_ = 0.0379, *wR*_2_ = 0.0585	*R*_1_ = 0.0409, *wR*_2_ = 0.0653
Final *R* indexes [all data]	*R*_1_ = 0.0726, *wR*_2_ = 0.1152	*R*_1_ = 0.0939, *wR*_2_ = 0.0883	*R*_1_ = 0.0693, *wR*_2_ = 0.0647	*R*_1_ = 0.0991, *wR*_2_ = 0.0804

To understand the effects of each structural component on the distortions and properties of quasi-2D perovskites, we first examine how structures differ as the perovskite layer thickness (*n*) increases (Figure 2A–C). In each of these structures, the 4FPEA spacer cation adopts a parallel slip-stacking arrangement within the interlayer, where the phenyl ring of one cation interacts strongly with the phenyl ring of a neighboring cation from the opposite layer via an offset π-π interaction (Figure S1). This slip-stacking arrangement is largely facilitated by the para-substitution of the polar fluorine atom and confers a highly rigid and interlocked organic interlayer, in contrast to the unfunctionalized PEA cation that is often edge-to-face stacked or disordered over multiple positions in the interlayer (Figure S1C). This offset π–π interlayer packing with 4FPEA is similar between the *n* = 1–3 phases but with some subtle differences.

The powder X-ray diffraction patterns (PXRD) of (4FPEA)_2_(MA)_*n*−1_Sn_*n*_I_3*n*+1_ (*n* = 1–3) (Figure 2E) exhibit periodic peaks every 5.35°, 3.90°, and 3.09° for *n* = 1, *n* = 2, and *n* = 3, which respectively correspond to reflections off their layered (*h*00) planes and agree with their respective layer thicknesses of 16.65 Å,^64^ 22.64 Å, and 28.88 Å. Thus, the out-of-plane stacking size increases by an average of 6.12 Å per layer of SnI_6_ octahedra as *n* increases. Interestingly, this increase in layer thickness is accompanied by a minor compression of the organic interlayer, where the interlayer spacing is initially 10.11 Å for *n* = 1, then reduced to 9.94 Å in *n* = 2, and finally to 9.88 Å in *n* = 3 (highlighted in Figure 2A–C). Since quasi-2D perovskites are balanced by the interplay of tensile strain from the inorganic sheets with the compressive strain from the spacer cations, this interlayer compression can be interpreted as an increase in the tensile strain from the thicker inorganic sheet, which is reminiscent of the behaviors of *n* = 2 structures that incorporate oversized A-cations.^19,40^ We therefore surmise that the ability to form higher *n* phases with a given spacer cation, as well as how thick the layer can become, is determined by how compressible the organic interlayer is as a result of the spacer cation structure. The reduced organic interlayer spacing and greater rigidity that arise from these higher *n* crystal structures might also account for the suppressed dynamic disorder of the spacer cations, reduced electron–phonon coupling, limited nonradiative recombination pathways, and improved carrier dynamics previously observed in quasi-2D perovskites in comparison to their corresponding *n* = 1 phases.^26,39,69,70^ Distinct and bright PL emission was observed at 627.2, 700.6, and 757.2 nm for the *n* = 1, *n* = 2, and *n* = 3 (4FPEA)_2_(MA)_*n*−1_Sn_*n*_I_3*n*+1_, respectively (Figure 2F), which display the expected redshifts as *n* increases. We note that the PL emission for the *n* = 2 phase is in the regime of wide bandgap perovskite absorbers for tandem solar cells,^71–73^ and the emission for the *n* = 3 phase is comparable to that of MAPbI_3_.^74–76^

We further assessed the variations in octahedral distortions (σ_1_^2^, σ_2_^2^ and *D*) between the different *n* phases of (4FPEA)_2_(A)_*n*−1_Sn_*n*_I_3*n*+1_, wherein minimized distortion favors increased metal-halide orbital overlap and therefore enhanced charge carrier properties.^42^ The *n* = 1 and *n* = 2 phases herein both have only one crystallographically unique octahedron, and the *n* = 3 phase has two unique octahedra corresponding to distinct outer versus inner octahedra within their inorganic trilayers (Figure 2D). In terms of interoctahedral distortions, as *n* increases, the in-plane Sn–I–Sn bond angle increases toward the ideal 180° (Table 2). The *n* = 2 and *n* = 3 phases also exhibit near-perfect to perfect 180° out-of-plane Sn–I–Sn bond angles, which is rare among quasi-2D RP perovskites, though sometimes observed in quasi-2D Dion-Jacobson perovskites.^50,77^ These ideal interoctahedral bond angles suggest minimal octahedral tilting and balancing of the vertical strain of the perovskite cage by 4FPEA, which we will further contextualize with other RP spacer cations in later sections.

**Table 2:** Comparisons of Structural Distortion Parameters between *n* = 1–3 (4FPEA)_2_(A)_*n*−1_Sn_*n*_I_3*n*+1_ Perovskites

Compound	(4FPEA)_2_SnI_4_ ^64^	(4FPEA)_2_(Cs)Sn_2_I_7_	(4FPEA)_2_(MA)Sn_2_I_7_	(4FPEA)_2_(FA)Sn_2_I_7_	(4FPEA)_2_(MA)_2_Sn_3_I_10_	
*n*	1	2	2	2	3	
					Inner	Outer
Sn–I–Sn (IP)/deg	156.37(1), 156.37(1)	157.32(3), 157.98(4)	159.01(4), 160.17(3)	159.43(2), 161.96(2)	162.70(3), 162.70(3)	159.90(3), 161.75(3)
Sn–I–Sn (OOP)/deg	-	180.00(6)	179.49(5)	180.00(1)	179.12(3)	
I–Sn–I (OOP)/deg	-	176.28(3)	176.48(3)	175.95(2)	180.00(4)	176.20(3)
Cage volume/Å^3^	-	238.39	244.38	251.82	244.33	
σ_1_^2^/deg^2^	3.82	4.55	3.71	5.30	0.11	4.49
σ_2_^2^/deg^2^	0.00	18.44	12.66	21.96	0.00	17.36
*D*	0.0064	0.0072	0.0116	0.0196	0.0019	0.0145
Interlayer spacing/Å^a^	10.114(1)	10.029(4)	9.942(3)	9.844(2)	9.882(1)	

a Measured as the distance between the mean planes of terminal iodide atoms for neighboring slabs.

In contrast to this reduction of interoctahedral distortion, the opposite trend is observed for the intraoctahedral distortion parameters (Figure 2G), which are generally low but increase slightly as *n* increases. Specifically, the *n* = 1 phase exhibits no off-centering of the metal center (σ_2_^2^ = 0), which is followed by minor increases in σ_2_^2^ and *D* for *n* = 2, then further increases for the outer octahedra of *n* = 3. The crystallographically distinct inner octahedra of the *n* = 3 structure exhibit minimal distortion (σ_1_^2^ = 0.11 and σ_2_^2^ = 0) compared to the more distorted outer layers. Sn–I–Sn bond angles close to 180° are also observed between the inner and outer layers of (4FPEA)_2_(MA)_2_Sn_3_I_10_, in contrast to the other known *n* = 3 structures that exhibit greater bond angle distortion.^12,21^ Thus, for the *n* = 3 inner octahedra, the compressive strain from the 4FPEA spacer cations and outer octahedral layers is perfectly balanced by the tensile strain from the MA cage cations to generate a highly symmetric bonding environment with low structural distortion, akin to 3D cubic perovskites. As such, quasi-2D perovskites (*n* ≥ 2) can possess not only suppressed interoctahedral distortion (i.e., octahedral tilting) but also potentially minimized intraoctahedral distortions (i.e., metal-site off-centering) through strain balancing with the appropriate spacer and A-cations.

### Cage Balancing of Different Spacer Cations

To further understand the origins of the minimized structural distortion of 4FPEA-based tin perovskites, we compare the (4FPEA)_2_(FA)­Sn_2_I_7_ structure against three other *n* = 2 (LA)_2_(FA)­Sn_2_I_7_ structures with different spacer cations with varying headgroup sterics and interlayer interactions compared to 4FPEA (Figure 3A–D), namely the butylammonium (BA), phenethylammonium (PEA), and 4-bromo-2-fluorobenzylammonium (BrFBZ) spacer cations.^19,20^ The fully aliphatic character of BA makes the ammonium headgroup less steric compared to 4FPEA, while the shorter tail and ortho-substituted fluorine of BrFBZ make the headgroup more steric. In addition, we report a new (PEA)_2_(FA)­Sn_2_I_7_ crystal structure (details in Table S2) to serve as an intermediate reference between the BA and 4FPEA structures.

**Figure 3:**
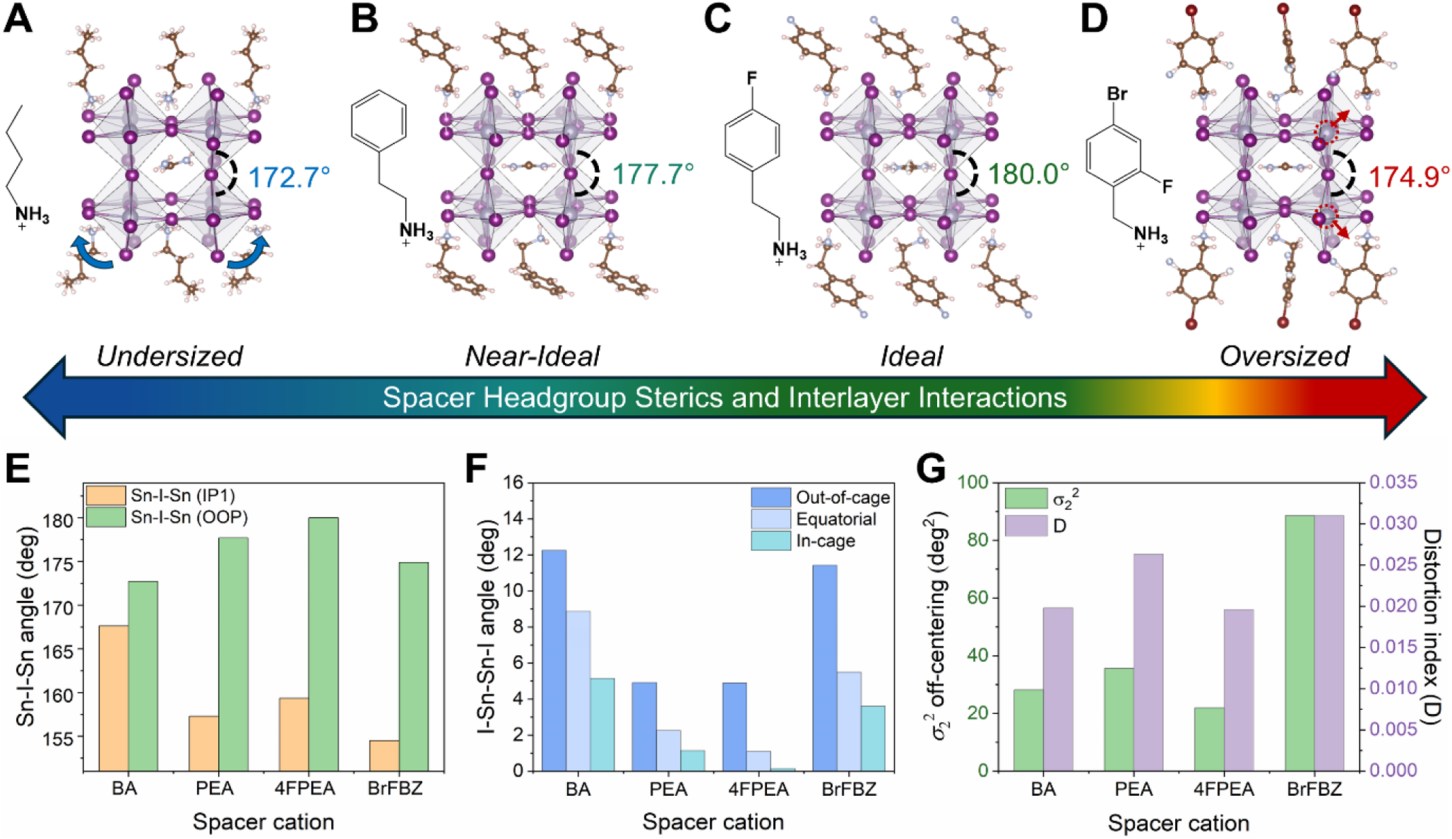
Impact of the spacer cation on structural distortion of *n* = 2 (LA)_2_(FA)­Sn_2_I_7_. Single crystal structures showing one perovskite cage unit of eight neighboring octahedra together with the corresponding spacer cations for (A) (BA)_2_(FA)­Sn_2_I_7_ [CIF adapted from ref 19. Copyright 2022 American Chemical Society], (B) (PEA)_2_(FA)­Sn_2_I_7_ (minor disorder components omitted for clarity), (C) (4FPEA)_2_(FA)­Sn_2_I_7_, and (D) (BrFBZ)_2_(FA)­Sn_2_I_7_ [CIF adapted from ref 20. Copyright 2023 American Chemical Society]. (E) In-plane and out-of-plane Sn–I–Sn bond angles, (F) I–Sn–Sn–I dihedral angles, and (G) σ_2_^2^ off-centering and bond distortion index (*D*) parameters for each of the four (LA)_2_(FA)­Sn_2_I_7_ structures.

Perhaps the most obvious structural impact on *n* = 2 perovskite cages by different spacer cations is the varied interoctahedral Sn–I–Sn angles that gauge the extent of octahedral tilting in the structure (Figure 3E). As the steric hindrance of the spacer cation headgroup increases, the out-of-plane Sn–I–Sn bond angle increases: 172.7° in the BA structure, 177.7° in the PEA structure, and a perfect 180.0° in the 4FPEA structure (highlighted on the respective crystal structures). Even though PEA and 4FPEA have the same headgroup, differences in the bond angles are observed for these two structures, suggesting that the interlayer interactions also play a key role in modulating the compressive strain imparted by the spacer cation.^59^ As the headgroup sterics further increase, steric strain on the inorganic network from the spacer cation subsequently distorts the bond angles, as shown by the 174.9° out-of-plane angle in the BrFBZ structure. These observations highlight the stark differences in the compressive strains exerted by different organic spacer cations, where compression of the perovskite cage from the BA cation is too weak and compression from the BrFBZ cation is too strong, leading to distortion in either case. Such observations concur with high pressure studies on 2D lead iodide perovskites with aliphatic spacer cations,^46,78^ in which the application of hydrostatic pressure enhances PL and photoconductivity up to a certain threshold (i.e., when the cage becomes balanced and metal-halide bonding is optimized) and then decreases due to overpressurization. Analogously, the different organic structures of the spacer cations can be regarded as a source of “chemical pressure” onto the inorganic network and 4FPEA seems to strike the right balance in its compressive effects compared to the other spacer cations.

In addition to the out-of-plane Sn–I–Sn angles, several other structural parameters, such as σ_1_^2^, σ_2_^2^, *D*, point to the minimal intraoctahedral distortion produced by 4FPEA over the other spacer cations (Table 3). The in-plane Sn–I–Sn bond angles are also improved in (4FPEA)_2_(FA)­Sn_2_I_7_ compared to the PEA and BrFBZ structures, but are interestingly the least distorted in the BA structure. This suggests some nuance in how the quasi-2D perovskite cages respond to the different compressive strains imparted by different spacer cations. To further understand these strain responses, we first focus on the I–Sn–Sn–I dihedral angles (Figure 3F), which further quantify the extent to which the SnI_6_ octahedra are rotated (i.e., tilted) relative to one another. In *n* = 2 perovskites, there exist three sets of dihedral angles that either face out-of-cage, equatorial (within the inorganic plane), or in-cage. The dihedral angles are the most severely distorted in the out-of-cage direction, intermediately distorted in the equatorial direction, and minimally distorted for the in-cage direction due to the extent to which each interacts with the spacer cation. This trend is general across these four structures and others previously reported.^19^ We found (4FPEA)_2_(FA)­Sn_2_I_7_ exhibits the smallest dihedral angles and therefore the least octahedral tilting among these four (LA)_2_(FA)­Sn_2_I_7_ structures. (BA)_2_(FA)­Sn_2_I_7_ exhibits the largest average dihedral angles across all directions, indicating greater octahedral tilting (highlighted in Figure 3A) is necessary to compensate for the lack of chemical pressure induced by the flexible BA cation. It is also through this tilting that the in-plane Sn–I–Sn bond angles of (BA)_2_(FA)­Sn_2_I_7_ structure could be slightly less distorted compared to (4FPEA)_2_(FA)­Sn_2_I_7_ despite having an overall more distorted structure. Interestingly, the dihedral angles (and thus octahedral tilting) are larger for (BrFBZ)_2_(FA)­Sn_2_I_7_, and its intraoctahedral distortions (σ_2_^2^ and *D*) are significantly higher compared to the other structures (Figure 3G). In particular, the σ_2_^2^ off-centering in (BrFBZ)_2_(FA)­Sn_2_I_7_ is over four times that of (4FPEA)_2_(FA)­Sn_2_I_7_, indicating that the metal site off-centering becomes more pronounced with overly steric spacer cation headgroups (highlighted with red arrows in Figure 3D). Both types of distortions present in quasi-2D perovskites, octahedral tilting (interoctahedral) and metal off-centering (intraoctahedral), depend on the structures and steric hindrance of the spacer cations. Notably, these strain responses through tilting and off-centering are analogous to the effects that undersized and oversized A-cations respectively have on 3D perovskites.^15,79,80^ Therefore, quasi-2D perovskites can similarly be optimized by selection of balanced combinations of spacer cations and A-cations. The example of (4FPEA)_2_(FA)­Sn_2_I_7_ illustrates that, by minimizing both the octahedral tilting and off-centering with the right cations, idealized bond angles and minimized distortion can be achieved.

**Table 3:** Comparisons of Select Structural Distortion Parameters between Different *n* = 2 (LA)_2_(A)­Sn_2_I_7_ Perovskites

Compound	(BA)_2_(FA)Sn_2_I_7_	(PEA)_2_(FA)Sn_2_I_7_	(4FPEA)_2_(FA)Sn_2_I_7_	(BrFBZ)_2_(FA)Sn_2_I_7_
Reference	^19^	This work	This work	^20^
Temperature/K	293.0	100.0	295.0	100.0
Cage Volume/Å^3^	259.34	245.64	251.82	249.55
Ave Sn–I bond length/Å	3.147	3.145	3.159	3.153
Sn–I–Sn (IP)/deg	168.80(1), 172.83(1), 167.62(1), 175.27(1)	157.28(5), 158.96(5), 161.23(4), 161.18(5)	159.43(2), 161.96(2)	154.51(3), 165.95(2)
Sn–I–Sn (OOP)/deg	172.72(6)	177.73(4)	180.00(1)	174.89(2)
σ_1_^2^/deg^2^	5.31	6.50	5.30	18.99
σ_2_^2^/deg^2^	28.19	35.57	21.96	88.59
*D*	0.0199	0.0263	0.0196	0.0311
Interlayer distance/Å^a^	7.174(1)	9.407(8)	9.844(2)	9.732(2)
Ave I–B–B–I dihedral angle (out-of-cage)/deg	12.26	4.92	4.89	11.43
Ave I–B–B–I dihedral angle (equatorial)/deg	8.87	2.26	1.10	5.50
Ave I–B–B–I dihedral angle (in-cage)/deg	5.14	1.14	0.15	3.61

a Measured as the distance between the mean planes of terminal iodide atoms for neighboring slabs.

### Air Stability of (4FPEA)_2_(A)_*n*−1_Sn_*n*_I_3*n*+1_

In addition to the impact of 4FPEA on structural distortion, the air stabilities of 4FPEA-based tin perovskites were investigated by tracking changes in the PXRD patterns of crushed single crystals as they aged in ambient air over 4 weeks (Figure 4). Compared to the benchmark (PEA)_2_SnI_4_ phase that almost fully degraded after 4 weeks (Figure 4A),^34^ each of the 4FPEA-based *n* = 1 and *n* = 2 tin perovskites exhibited less new peak growth and therefore less oxidation over time, which attests to the protective capabilities of 4FPEA. Within 4 weeks, no apparent changes were observed in the PXRD patterns for (4FPEA)_2_SnI_4_ (Figure 4B) and only minor changes for (4FPEA)_2_(FA)­Sn_2_I_7_ (Figure 4C). Similarly, no changes were observed for (4FPEA)_2_(Cs)­Sn_2_I_7_ (Figure S2A). More apparent changes are observed in the PXRD pattern of (4FPEA)_2_(MA)­Sn_2_I_7_, for which the original (*h*00) reflections were still present, but several new peaks with considerable intensity emerged after 4 weeks (Figure S2B). Although better protection by 4FPEA significantly suppresses the oxidation of (4FPEA)_2_(MA)­Sn_2_I_7_ compared to (PEA)_2_SnI_4_, the more volatile nature of the MA cation could accelerate the degradation of the perovskite, as suggested by detailed studies of (A)­PbI_3_ perovskites.^81^ These show that the protective capabilities of 4FPEA can extend well into quasi-2D phases, but the air stability also depends on the A-cation.

**Figure 4:**
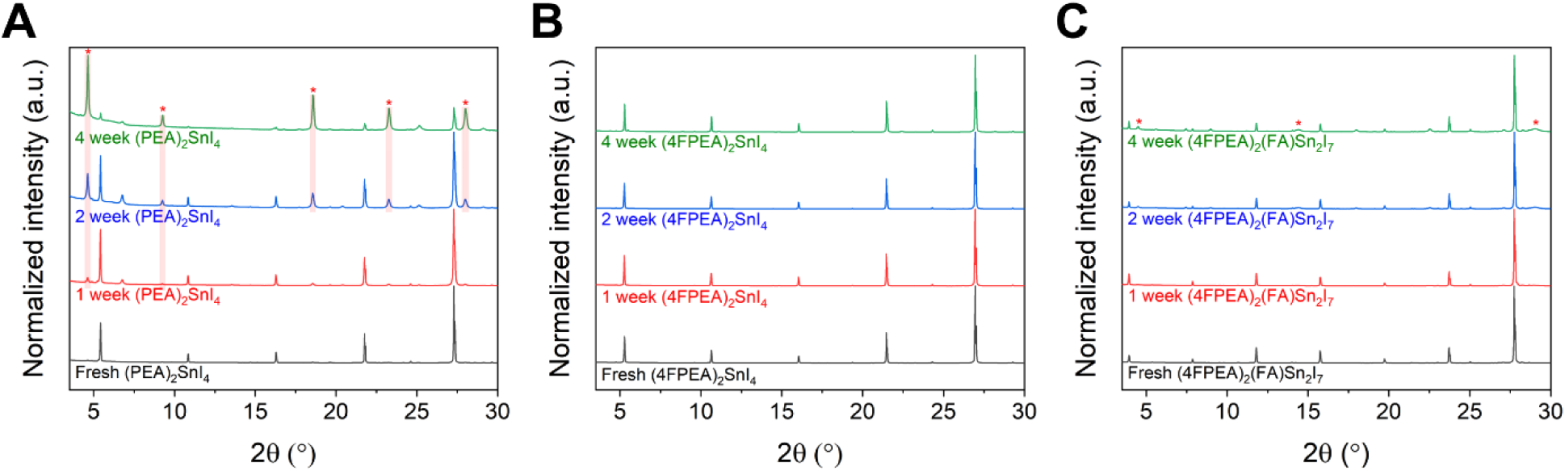
Air stability of 4FPEA-based 2D tin perovskites assessed by PXRD. PXRD patterns over 4 weeks of ambient air exposure are shown for (A) PEA_2_SnI_4_ (adapted from ref 34), (B) 4FPEA_2_SnI_4_, and (C) (4FPEA)_2_(FA)­Sn_2_I_7_. Red highlights and asterisks indicate the growth of non-(*h*00) reflections corresponding to degradation products. All data were taken on crushed single crystals aged in ambient air with RH = 20–45%.

### Balancing Effects of the A-Site Cations and Photophysical Correlations

Having established the compressive effects of 4FPEA and other spacer cations, we next investigated the tensile effects from the A-site cations on (4FPEA)_2_(A)­Sn_2_I_7_ structures (A = Cs^+^, MA, FA) and their correlation with PL properties. While Cs^+^ incorporation in *bromide* perovskites is more common,^82–84^ (4FPEA)_2_(Cs)­Sn_2_I_7_ is a rare example of a quasi-2D lead or tin-based *iodide* perovskite crystal structure that incorporates the smaller Cs^+^ cation. Previous studies have suggested that the phase formation of lead or tin-based (LA)_2_(Cs)­B_2_I_7_ was either entirely unfavorable with some spacer cations or was possible but resulted in poor crystallinity with other spacer cations.^19,20,85^ Thus, the strongly balancing character of 4FPEA, templating effects, and solubility must be conducive to Cs^+^ incorporation and high-quality single crystal growth. The synthesis of (4FPEA)_2_(A)­Sn_2_I_7_ with oversized A-cations was attempted, but no evidence of *n* > 1 phase formation was found (Figure S3).

Significantly, each (4FPEA)_2_(A)­Sn_2_I_7_ structure with A = Cs^+^, MA, FA (Figure 5A–C) exhibits nearly identical out-of-plane Sn–I–Sn bond angles close to 180.0°, suggesting similar compression from 4FPEA and “balanced” cages in each structure. Meanwhile, in-plane Sn–I–Sn angles and *D* increase as the A-cation size increases from Cs^+^ to MA to FA due to the cage expansion from larger A-cations (values in Table 2). In contrast, the intraoctahedral distortions, described by the bond angle variance parameters (σ_2_^2^), do not follow a monotonic trend with the increasing size of the A-cations (Figure 5D). Specifically, the I–Sn–I bond angles are closest to the ideal 90° and 180° in the MA-based structure and the most extreme I–Sn–I angles for each structure are highlighted in the insets of Figure 5A–C. The smallest σ_2_^2^ value is observed in (4FPEA)_2_(MA)­Sn_2_I_7_, which is followed by (4FPEA)_2_(Cs)­Sn_2_I_7_, then (4FPEA)_2_(FA)­Sn_2_I_7_.

**Figure 5:**
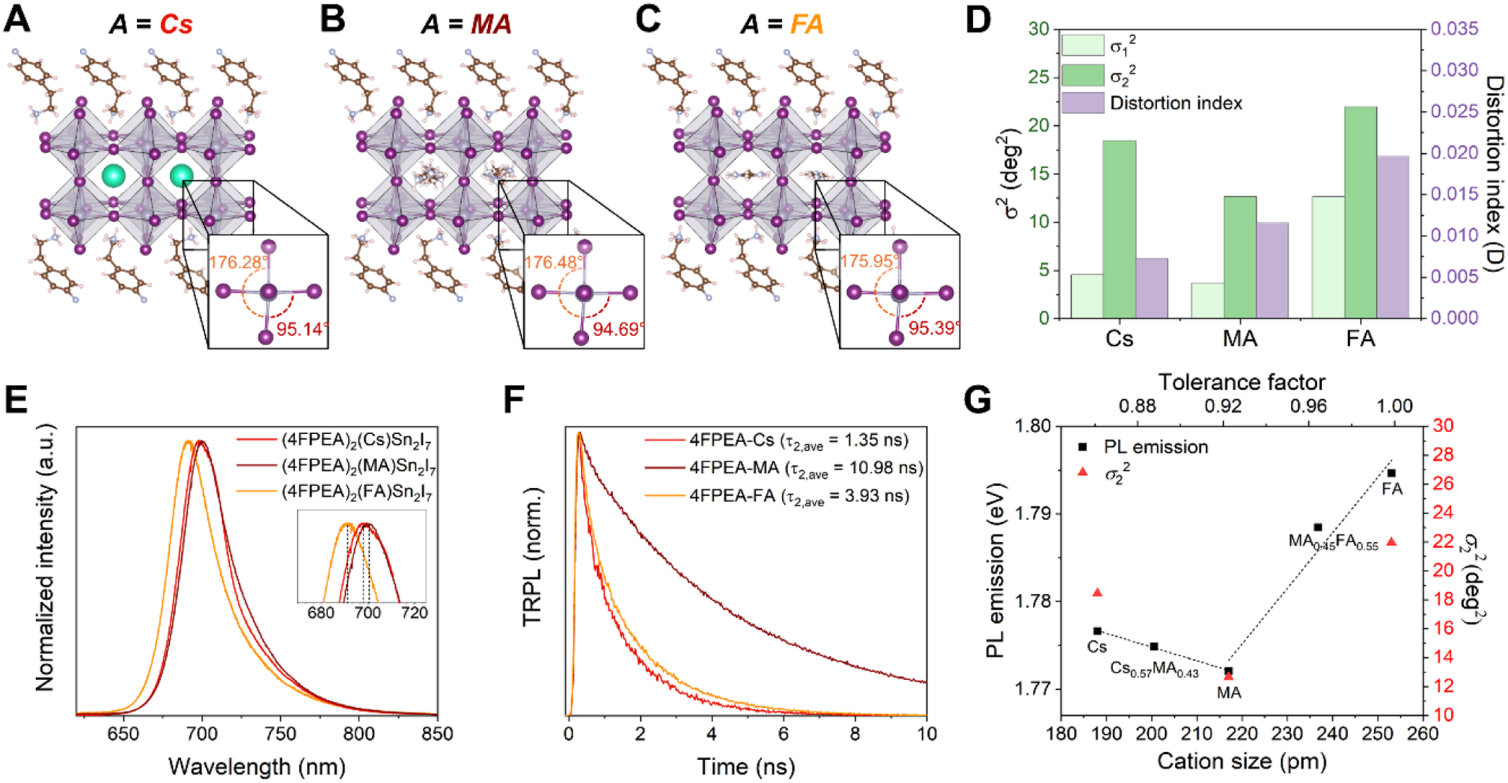
Impacts of the A-site cation on structural distortion and PL properties of *n* = 2 (4FPEA)_2_(A)­Sn_2_I_7_. Single crystal structures for (A) (4FPEA)_2_(Cs)­Sn_2_I_7_, (B) (4FPEA)_2_(MA)­Sn_2_I_7_, and (C) (4FPEA)_2_(FA)­Sn_2_I_7_, with insets showing the most extreme I–Sn–I bond angles in each structure. (D) Bond angle variances (σ_1_^2^ and σ_2_^2^) and bond distortion indices (*D*), (E) PL spectra, and (F) time-resolved PL spectra for the three (4FPEA)_2_(A)­Sn_2_I_7_. (G) Correlations between the PL properties and structural distortion (specifically σ_2_^2^ value) of (4FPEA)_2_(A)­Sn_2_I_7_ with the A-cation size.

To understand the correlation of the structural distortions with optoelectronic properties, we examined the PL spectra of (4FPEA)_2_(A)­Sn_2_I_7_ (Figures 5E and S4A–C). Sharp PL emission was observed at 697.1 ± 0.7 nm for the Cs phase, 700.6 ± 0.9 nm for the MA phase, and 690.3 ± 0.5 nm for the FA phase. This trend in red-shifted PL emission for (4FPEA)_2_(MA)­Sn_2_I_7_ followed by (4FPEA)_2_(Cs)­Sn_2_I_7_, then (4FPEA)_2_(FA)­Sn_2_I_7_ correlates well with the trend in σ_2_^2^ off-centering and the emissive characteristics found in 3D (A)­SnI_3_ perovskites.^15^ Similarly, the MA phase also exhibits the longest average radiative lifetimes compared to the Cs and FA phases, with representative time-resolved photoluminescence (TRPL) spectra shown in Figure 5F (and full data and statistics provided in Figures S5–S8 and Table S3). Interestingly, it is the Cs phase that exhibits the shortest TRPL lifetimes, and not the FA phase with a larger σ_2_^2^. This behavior agrees with a recent report of faster hot-carrier cooling in the fully inorganic 3D CsSnI_3_ versus the hybrid 3D (A)­SnI_3_ perovskites (MASnI_3_ and FASnI_3_),^86^ suggesting that there may be similarities in carrier relaxation pathways between 3D and quasi-2D perovskites according to the A-site cations.

We found a strong correlation between the PL emission energy with the octahedral distortion in the (4FPEA)_2_(A)­Sn_2_I_7_ with different A-cation sizes (Figure 5G). To further supplement this trend of PL emission wavelength with A-cation size, we synthesized alloyed phases of (4FPEA)_2_(Cs_0.57_MA_0.43_)­Sn_2_I_7_ and (4FPEA)_2_(MA_0.45_FA_0.55_)­Sn_2_I_7_ (details in Table S1), which can be treated as linear combinations of their respective end phases. The relative ratios between the A-cations in these compounds were determined via ICP-OES (Figure S9 and Table S4) and ^1^H NMR (Figure S10), respectively. The PL emission wavelength (i.e., optical bandgap) of these two alloyed (4FPEA)_2_(A)­Sn_2_I_7_ fell between the emission wavelengths of their respective end phases (Figures 5G and S4D–F). This bandgap “bowing effect” in quasi-2D tin perovskites with more oversized A-cations has been observed in BA_2_(A)­Sn_2_I_7_,^19^ and could be attributed to the smaller metal site off-centering (σ_2_^2^) in the MA structure compared to the Cs and FA phases, despite their similar bond angles. These insights suggest that further property tuning is possible using A-site cations.

### Amplified Spontaneous Emission in (4FPEA)_2_(A)_*n*−1_Sn_*n*_I_3*n*+1_

To further investigate the optoelectronic performance of (4FPEA)_2_(A)_*n*−1_Sn_*n*_I_3*n*+1_, we examined the amplified spontaneous emission (ASE) behaviors of the exfoliated flakes (experimental details in the SI). Both ASE and lasing share the mechanism of optical amplification through stimulated emission, but differ fundamentally in coherence properties and emission characteristics. ASE occurs when spontaneous emission is amplified via stimulated emission. Unlike lasing, ASE does not require an optical cavity nor resonator; as a result, the emission is noncoherent and broadband compared to lasing, reflecting the gain spectrum that corresponds to the excited states which have achieved population inversion. Lasing only occurs when an optical resonator is present. Lasing arises exclusively when there is spectral overlap between the optical cold cavity mode (which satisfies the standing wave condition) and the material’s gain spectrum. Since ASE is not affected by the coupling with an optical resonator, it provides a better insight into the intrinsic properties of the material governing the realization of population inversion. In general, for ASE to occur in a semiconducting material, the optical gain from stimulated emission must exceed any nonradiative losses after some critical excitation threshold is reached, which stipulates excellent light emission, robust excitons, and low trapping from deep defects.^87–90^ However, ASE and lasing have remained uncommon among 2D tin and lead iodide perovskites.^25,26,28^ Notably, each of the 4FPEA-based 2D tin perovskites exhibited sharp ASE at 77 K (Figure 6A). The mechanical exfoliation process can produce variable microcrystal size and morphology and therefore differences in ASE thresholds between different objects.^26,91^ The minimum ASE threshold values found for each (4FPEA)_2_(A)_*n*−1_Sn_*n*_I_3*n*+1_ phase, as well as the (PEA)_2_(FA)­Sn_2_I_7_ phase as a comparison, are shown in Figure 6B. They represent the lower boundaries for ASE among the surveyed objects. Representative examples of the fluence-dependent PL spectra and threshold determinations for (4FPEA)_2_(MA)­Sn_2_I_7_ and (4FPEA)_2_(MA)_2_Sn_3_I_10_ are shown in Figure 6C–F, respectively. A more complete data set collected on additional microflakes for each (4FPEA)_2_(A)_*n*−1_Sn_*n*_I_3*n*+1_ and (PEA)_2_(FA)­Sn_2_I_7_ is shown in Figures S11–S16.

**Figure 6:**
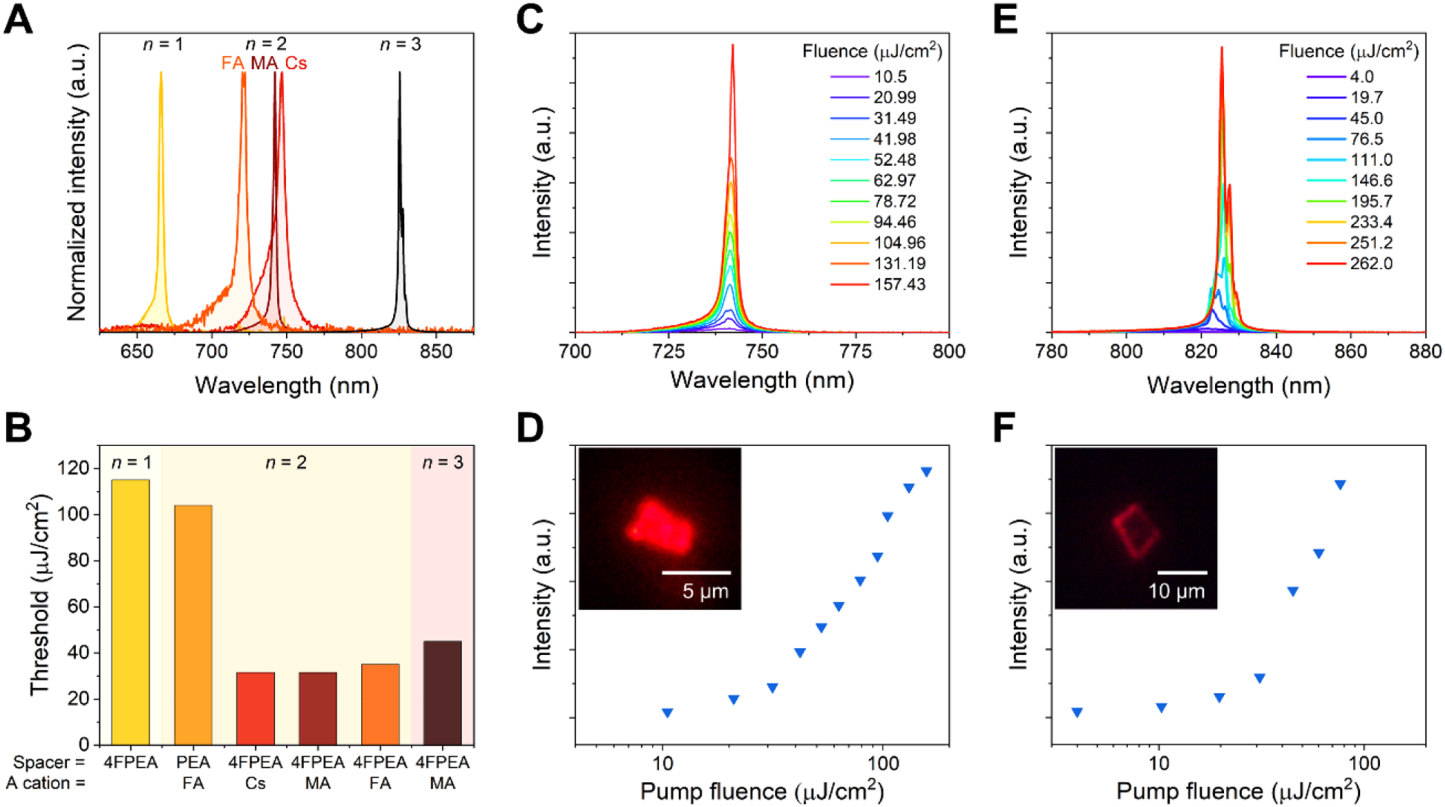
Amplified spontaneous emission in microflakes of (4FPEA)_2_(A)_*n*−1_Sn_*n*_I_3*n*+1_. (A) Representative ASE spectra at 77 K are shown for (4FPEA)_2_SnI_4_ at 173 μJ/cm^2^ fluence, (4FPEA)_2_(Cs)­Sn_2_I_7_ at 152 μJ/cm^2^ fluence, (4FPEA)_2_(MA)­Sn_2_I_7_ at 157 μJ/cm^2^ fluence, (4FPEA)_2_(FA)­Sn_2_I_7_ at 89 μJ/cm^2^ fluence, and (4FPEA)_2_(MA)_2_Sn_3_I_10_ at 257 μJ/cm^2^ fluence. (B) Dependence of the ASE thresholds on layer thickness (*n*), spacer cation, and A-site cation for the new 4FPEA (and PEA) quasi-2D perovskites reported in this work. (C) PL spectra and (D) PL intensity as a function of fluence for a representative microflake of (4FPEA)_2_(MA)­Sn_2_I_7_. (E) PL spectra and (F) PL intensity as a function of fluence for a representative microflake of (4FPEA)_2_(MA)_2_Sn_3_I_10_. The insets of panels (D,F) display the optical images of the crystals under laser excitation.

Among the 4FPEA series, the *n* ≥ 2 phases exhibit lower minimum ASE thresholds compared to the *n* = 1 (4FPEA)_2_SnI_4_, similar to the trend recently reported on a different 2D Sn perovskite series,^91^ and each of the (4FPEA)_2_(A)­Sn_2_I_7_ with different A-cations exhibited similar minimum thresholds among the surveyed objects. Interestingly, the thresholds for (4FPEA)_2_(A)­Sn_2_I_7_ were also lower than that of (PEA)_2_(FA)­Sn_2_I_7_, which might be attributed to their more favorable structural distortions discussed above alongside possible contributions from the enhanced dielectric screening caused by the stronger dipoles in 4FPEA.^65^ Moreover, no ASE was observed across pump fluence for the previously reported (BrFBZ)_2_(FA)­Sn_2_I_7_ with more severe structural distortions (see Figure 3D),^20^ which may further suggest the importance of minimizing structural distortion and cage balancing to improve the photophysics and obtain ASE in quasi-2D tin perovskites. In addition to the achievement of ASE in each of the 4FPEA-based perovskites, we observed multimode lasing in a randomly exfoliated microflake of (4FPEA)_2_(MA)_2_Sn_3_I_10_ (Figure 6E,F). The microcrystal exhibited sharp and narrow emission localized at the crystal edges (see Figure 6F inset for an optical image under excitation), suggestive of whispering gallery mode (WGM) lasing and optical confinement due to the high crystallinity and favorable morphology of the exfoliated mircrocrystal.^91,92^ In general, the lasing thresholds for (4FPEA)_2_(A)_*n*−1_Sn_*n*_I_3*n*+1_ fall into the same regime as those previously reported for other 2D perovskite crystals and flakes,^26,27^ which span a range from about 2 to 180 μJ/cm^2^. However, it should be noted that a direct comparison of the lasing performance of 2D perovskites is not straightforward, as the lasing threshold depends on several factors, including the sample geometry, thickness, and roughness, as well as the excitation conditions (pump energy and laser pulse width) and temperature, which vary across different studies. Therefore, these threshold values primarily serve as indicators of the material’s potential as coherent light emitters, but should not be rigorously used as absolute figures of merit for comparison. Nevertheless, the promising ASE and lasing in (4FPEA)_2_(A)_*n*−1_Sn_*n*_I_3*n*+1_ serves as a testament to their excellent photophysics, which, when coupled with their excellent air stability, could readily lend them toward robust high-performance optoelectronic applications.

### Classifications of Structural Distortions and Perovskite Cages in Quasi-2D Perovskites

The (4FPEA)_2_(A)_*n*−1_Sn_*n*_I_3*n*–1_ structures studied herein illustrate the importance of synergistic structural tuning for better photophysical properties and serve as a nice case study. To further contextualize our findings and better understand the structural origins for minimized distortion and improved photophysics in quasi-2D perovskites, we performed a comprehensive survey of over 60 published *n* = 2 (both lead and tin) iodide RP perovskite structures (Figure 7). As described in Figure 1, the crystal structures become increasingly more complex with quasi-2D perovskites. At the BX_6_ octahedron level, the strain introduced by the organic cations can result in either ideal, angularly distorted, or off-centered octahedra, which are classified by the intraoctahedral σ_1_^2^ and σ_2_^2^ parameters (Figure 7A). The assembly of these building blocks into 2D *n* = 1 layers of corner-sharing BX_6_ octahedra introduces interoctahedral distortion parameters such as interoctahedral in-plane B–I–B bond angles and I–B–B–I dihedral angles. The further buildup to bilayer networks of BX_6_ octahedra (*n* = 2) introduces another interoctahedral distortion parameter, the out-of-plane B–I–B bond angle. Therefore, in quasi-2D *n* = 2 (LA)_2_(A)­B_2_I_7_ perovskites, the intraoctahedral distortions (σ_1_^2^ and σ_2_^2^), interoctahedral distortions (various B–I–B angles and dihedral angles), and cage volume and geometry are all modulated by the interplay of both the spacer and A-site cations, which make them more difficult to fully understand.^42^ Below, we describe the different types of distorted cage structures elucidated by our survey and reveal the trends we found.

**Figure 7:**
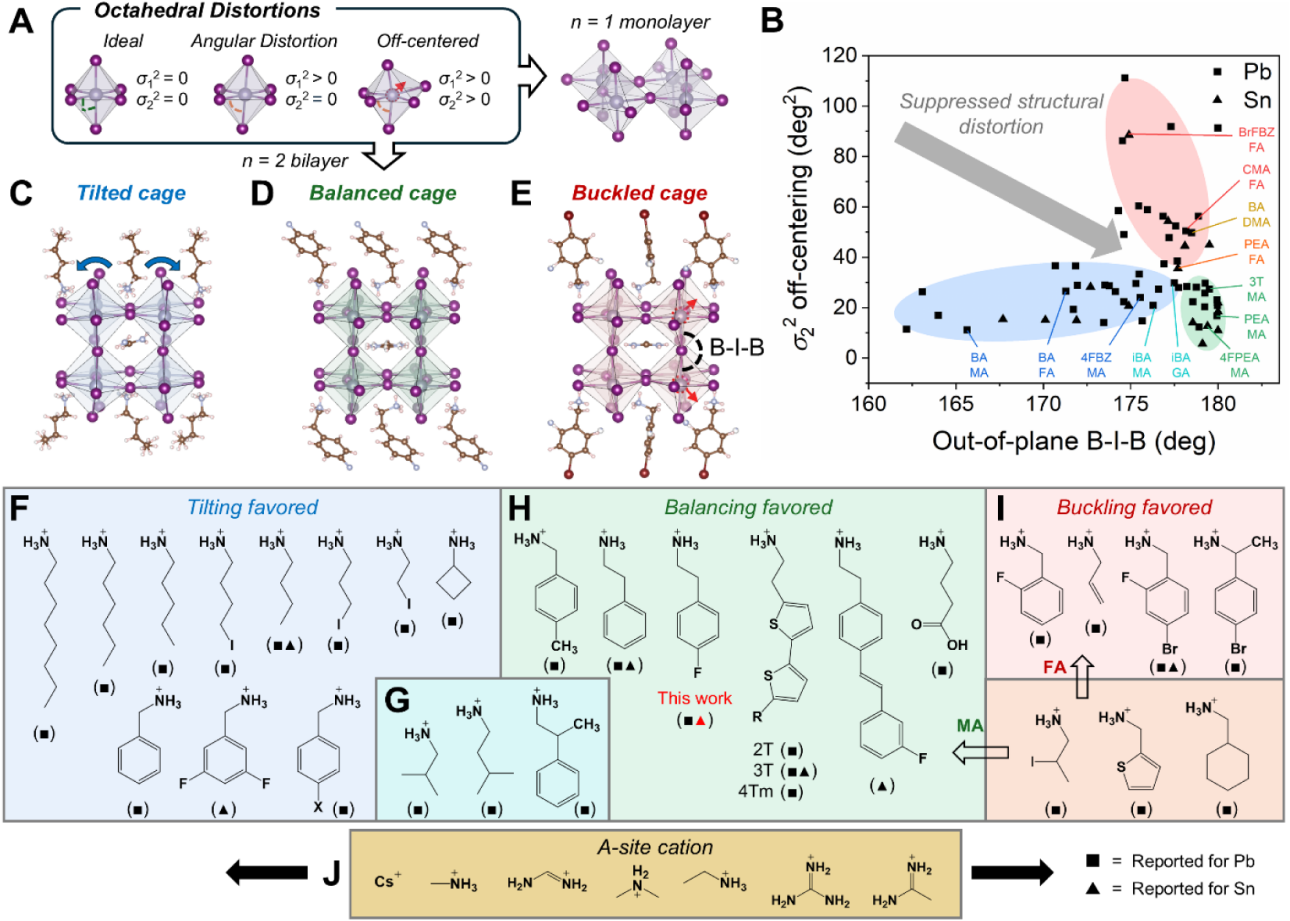
Classifications of quasi-2D *n* = 2 RP iodide perovskites based on perovskite cage type. (A) Different octahedral distortions and their corresponding bond angle variance parameters, σ_1_^2^ and σ_2_^2^. Using these BX_6_ octahedra, *n* = 1 monolayer and *n* = 2 2D perovskite networks can be built. (B) Classification of over 60 reported *n* = 2 lead (square symbol) and tin (triangle symbol) iodide perovskites displayed based on their σ_2_^2^ off-centering parameter and out-of-plane B–I–B angle into three distinct structural regimes that are guided visually by blue, green, and red ovals. All values are determined from reported crystal structures (tabulated in Table S5), and average values were used when multiple unique octahedra or angles were present. The three major classes of quasi-2D perovskite cages found: (C) tilted perovskite cage, such as that found in (BA)_2_(FA)­Sn_2_I_7_ (blue region in panel B), (D) balanced cage, such as that in (4FPEA)_2_(FA)­Sn_2_I_7_ (green region in panel B), and (E) buckled cage, such as that in (BrFBZ)_2_(FA)­Sn_2_I_7_ (red region in panel B). Spacer cations that favor (F) tilting, (G) near-balanced with minor tilting, (H) cage balancing, and (I) buckling for the perovskite structures surveyed in panel B, and the cage types could be further altered with the selection of (J) various A-site cations with increasing radii. Reported Pb- and Sn-based structures are denoted with squares and triangles, respectively.

Through a systematic survey of >60 *n* = 2 RP lead and tin iodide perovskite crystal structures reported to date (Table S5) and observing any clustering behaviors that emerge among the various structural parameters (Figure S17), we found three distinct classes of *n* = 2 perovskite cages that can be identified based on an 2D-plot of out-of-plane B–I–B bond angle distortion and σ_2_^2^ off-centering (Figure 7B). We name these three distinct classes of *n* = 2 perovskite cages as [1] “tilted” cages, in which interoctahedral (out-of-plane B–I–B) distortions are present but intraoctahedral distortions (σ_2_^2^) are low (Figure 7C); [2] “balanced” cages, in which both interoctahedral and intraoctahedral distortions are suppressed (Figure 7D); and [3] “buckled” cages, in which both interoctahedral and intraoctahedral distortions are present (Figure 7E). These three distinct structural regimes naturally emerge as clusters of data points in Figure 7B, as highlighted in blue, green, and red for the tilted, balanced, and buckled cages, respectively. Such clustering emerges in this 2D plot due to the structural parameters: each tilted phase is characterized by low σ_2_^2^ but out-of-plane B–I–B angles significantly less than 180° (and large dihedral angles), leading to cages built from octahedra with minimal off-centering but substantial interoctahedral tilting (Figure 7C); each buckled phase is characterized by similarly nonideal out-of-plane B–I–B angles (and large dihedral angles), but also much larger σ_2_^2^ values, leading to highly distorted cages that are built from misaligned octahedra with large metal cation off-centering (hence called “buckled”) (Figure 7E). Lastly, each balanced phase is characterized by minimal off-centering (low σ_2_^2^) and near-180° out-of-plane B–I–B angles, leading to cages built with ideal alignment of nearly perfect octahedra (Figure 7D). As with the sorting scheme shown in Figure 7B, similar clustering can be observed with structural distortion parameters σ_1_^2^, dihedral angles, and in-plane B–I–B angles but with weaker separation between some cage classes (Figure S17B–D). For instance, both tilted and buckled cages exhibit larger I–B–B–I dihedral angle distortion. Interestingly, even though B–I bond distances do not strongly correlate across the cage classes (Figure S17E,F), balanced cages generally exhibit smaller cage volumes compared to tilted and buckled cages (Figure S17G,H). However, plotting other structural parameters such as bond distortion index *D*, NH_3_ penetration, spacer volume and others, revealed no apparent clustering behaviors (Figure S17I–L).

Intuitively, both B–I–B bond angle distortions and octahedral off-centering of the metal site reduce the overlap of the metal and halide *s* and *p* orbitals responsible for the semiconducting properties of these materials.^42,55^ Therefore, the classification scheme introduced in Figure 7B maps how effectively these distortions could be suppressed, which also correlates with the enhancement of photophysical properties. One might expect that the 2D perovskite structures located closer to the lower right corner of this plot would have more balanced cages and thus good optoelectronic properties. Indeed, many of the reported quasi-2D (both lead and tin) perovskite structures with high-performance applications lie in this balanced cage regime with minimized distortion (highlighted by the green oval in Figure 7B). Most notably are the *n* = 2 (PEA)_2_(MA)­Pb_2_I_7_ that are commonly applied as quasi-2D passivation and charge funneling layers in perovskite solar cells,^93,94^ (3T)_2_(MA)­B_2_I_7_ and similar oligothiophene-based *n* = 2 structures favorable for light emission and lasing,^26,28,95^ and now the new *n* = 2 (4FPEA)_2_(A)­Sn_2_I_7_ perovskites capable of ASE that remain balanced with Cs^+^, MA, and FA cations. This classification scheme also agrees well with the general performance trends among 3D APbI_3_ perovskites, with α-FAPbI_3_ lying in the ideal balanced regime compared to less desirable polymorphs (Figure S18).

Figure 7F–I displays the spacer cations that favor tilting, balancing, buckling, and the cases in between, in these reported *n* = 2 iodide perovskites surveyed in Figure 7B, with key examples denoted. Many of the spacer cations that favor tilting of the perovskite cage are aliphatic, much like the prototypical BA case (Figure 7F). However, benzylammonium and its meta- and para-functionalized derivatives, such as 4FBZ (4-fluorobenzylammonium), are also found to favor tilted cages due to their comparatively weaker interlayer interactions and different packing motifs (likely due to the halogenation, as illustrated in Figure S19A–C). Thus, cage tilting generally ensues from spacer cations that are insufficiently steric, have weak interlayer interactions, or both. This trend is further corroborated with the examples of spacer cations that induce only minor tilting of the *n* = 2 perovskite cage and provide near-balanced structures (Figure 7G). In particular, branching of the aliphatic chains like in isobutylammonium (iBA) can increase the steric strain from aliphatic spacers in (iBA)_2_(MA)­Pb_2_I_7_, and the branching off of the β-carbon in β-methylphenethylammonium (β-MPEA) inhibits the interlayer π-π interactions in (β-MPEA)_2_(MA)­Pb_2_I_7_ compared to unfunctionalized PEA (Figure S19D).

In contrast to the spacer cations that lead to tilting, most spacer cations that favor balanced cages are like 4FPEA (Figure 7H). That is, each cation either features an ethylammonium tail off a bulkier aromatic group, exhibits strong interlayer interactions (such as π-π stacking or H-bonding), or both. Given the ubiquity of this character in producing balanced *n* = 2 cages, the compressive strains imparted by these spacer cations must similarly be in a “goldilocks” regime that is neither too weak to cause cage tilting nor too strong to cause cage buckling. In contrast, the spacer cations that favor buckling almost always exhibit sterically hindered ammonium headgroups (Figure 7I). This behavior is most clearly visible with the BrFBZ spacer cation discussed above (Figure 3D) and 2-fluorobenzylammonium (2FBZ) spacer cation that both have sterically hindered headgroups due to the ortho-substitution,^20,96^ or with the 4BrMBA spacer cation (R/S-4-bromo-α-methylbenzylammonium), for which the branching from the α-carbon imparts higher steric strain. Steric spacer cations with these characteristics tend to result in buckled cages and suitable structural distortions that lead to noncentrosymmetric quasi-2D perovskites useful for ferroelectric and spin–orbitronic applications.^96^

Beyond the clear impacts that spacer cations have on cage class, the A-cation (Figure 7J) has further contributions that, in some cases, can either enhance or reduce structural strains and shift the perovskite cage from one class to another (i.e., influence cage balancing). Some examples are highlighted in the lower panel of Figure 7I, where *n* = 2 RP perovskites based on the 2IPrA (2-iodopropylammonium), ThMA (thiophenemethylammonium), or CMA (cyclohexanemethylammonium) spacer cations adopt balanced cages with MA, but buckled cages with FA. In these cases, the use of the smaller MA cation is favorable in reducing the off-centering of the structure and achieving more balanced cages. Moreover, in certain tilted cages based on aliphatic spacer cations that can incorporate oversized A-cations (i.e., those larger than FA: dimethylammonium (DMA), ethylammonium (EA), guanidinium (GA), and acetamidinium (AA)),^19,41,97^ the oversized A-cations can sometimes improve the out-of-plane B–I–B bond angles, albeit at the cost of greater σ_2_^2^ off-centering, illustrated by the (BA)_2_(A)­Pb_2_I_7_ series denoted in Figure 7B. Specifically, (BA)_2_(DMA)­Pb_2_I_7_ exhibits elevated σ_2_^2^ values but near-180° out-of-plane Pb–I–Pb angles. This suggests that there might be unexplored combinations of spacer cations with oversized A-cations that could balance the *n* = 2 cage, as achieved with 4FPEA-like spacers paired with conventional A-cations. However, A-cation “size” alone might not account for the influence of unconventional large A-cations, and their shape and hydrogen bonding behaviors also need to be considered.^41^ These examples clearly show that synergistic interplay of both the spacer cations and A-cations is important for designing balanced perovskite cages (or other desired cage types) and tuning physical properties in quasi-2D perovskites.

## Conclusion

In summary, we report four new *n* = 2 and *n* = 3 quasi-2D tin iodide perovskite structures based on the fluorinated 4FPEA spacer cation and three different A-site cations (A = Cs^+^, MA, FA) that each exhibit excellent air stability and photophysical properties. In-depth crystal structure analysis elucidated the effects of layer thickness, spacer cation, and A-cation on the structures and optoelectronic properties of these quasi-2D tin iodide perovskites. The (4FPEA)_2_(A)­Sn_2_I_7_ perovskites exhibit both minimal intraoctahedral distortion (with minimal metal off-centering) and minimal interoctahedral distortion (undistorted 180° out-of-plane Sn–I–Sn bond angles), different from other *n* = 2 perovskite structures formed with other spacer cations. Moreover, the A-cation has subtle influence on structural distortions and notable impacts on optical properties. ASE was observed for each of the 4FPEA-based 2D tin perovskites at 77 K, along with lasing in microflakes of (4FPEA)_2_(MA)­Sn_3_I_10_. Based on a comprehensive structure survey, we developed a structural analysis method that can classify the reported *n* = 2 perovskite structures based on their structural distortion parameters that describe both intraoctahedral and interoctahedral distortions into one of three perovskite cage types: tilted, balanced, or buckled. Notably, in addition to the high air stability enabled by 4FPEA, (4FPEA)_2_(A)­Sn_2_I_7_ perovskites are among a handful of lead and tin iodide perovskites with well-balanced cages that have demonstrated high-performance optoelectronic applications. The structural insights and the cage balancing approach developed herein further motivate the rational design of previously less explored but more complex quasi-2D perovskites through the synergy of the spacer cation and A-cation. This will pave the way for discovering new quasi-2D (lead-free) halide perovskites for practical applications in high-performance optoelectronics.

## Supplementary Material



## Notes

Materials and methods, crystal structure refinement data, additional PXRD and PL data, compiled TRPL spectra, ICP-OES calibration curves, ^1^H NMR data, and compiled fluence-dependent PL, ASE data, and microscope images of (4FPEA)_2_(A)_*n*−1_Sn_*n*_I_3*n*+1_ exfoliated microflakes, compiled structure analyses and distortion calculations of reported quasi-2D iodide perovskites, crystal structure comparisons (PDF)
