# Supplementary material for: Cage Balancing Enhances Optoelectronic and Lasing Performance in Stable Quasi-2D Tin Iodide Perovskites
Source: J Am Chem Soc. 2025 Sep 15;147(38):34706–20. doi: 10.1021/jacs.5c09938 (PMC12503365; doi:10.1021/jacs.5c09938)
Supplement: Supplementary file 1 [file ja5c09938_si_001.pdf]

Supporting Information for

Cage Balancing Enhances Optoelectronic and Lasing Performance in Stable Quasi-2D Tin Iodide Perovskites

Christopher T. Triggs,[1] Chun-Sheng Jack Wu,[2] Yarong He,[2] Eliana Bernat,[1] Willa Mihalyi-Koch,[1] Kristel M. Forlano,[1] Ilia A. Guzei,[1] Daniele Cortecchia,[2,3] Annamaria Petrozza,[2*] Song Jin[1*]*

[1] Department of Chemistry, University of Wisconsin-Madison, Madison, WI 53703, United States

[2] Center for Nanoscience and Technology, Instituto Italiano di Tecnologia, via Rubattino 81, 20134 Milano, Italy.

[3] Department of Industrial Chemistry Toso Montanari, University of Bologna, via Piero Gobetti 85, 40129 Bologna, Italy

[*] Email: jin@chem.wisc.edu, annamaria.petrozza@iit.it, daniele.cortecchia2@unibo.iit

Contents:

Materials and Methods

Supporting Tables S1-S5

Supporting Figures S1-S19

References

Materials and Methods

Tin(II) chloride dihydrate (Sigma Aldrich, 98% ACS reagent), 4-fluorophenethylamine (4FPEA; TCI, >98%), phenethylammonium (PEA) iodide (GreatCell Solar), cesium iodide (Sigma Aldrich, 99.9%), methylammonium iodide (Sigma Aldrich), formamidinium iodide (GreatCell Solar), hydroiodic acid ≥47.0% with ≤1.5% hypophosphorous acid stabilizer (Sigma Aldrich), and hypophosphorous acid 50% (Sigma Aldrich) were used as received.

Large single crystals of $(4FPEA)_2(A)_{n-1}Sn_nI_{3n+1}$ (n = 1-3, A = Cs^+, MA, FA) and $(PEA)_2(FA)Sn_2I_7$ were grown via the slow-cooling method in hydroiodic acid according to the recipes of precursors provided in Table S1 following the procedures described below. The use of off-stoichiometric ratios was necessary to drive phase-pure $n = 2$ formation, which is typical in the synthesis of quasi-2D perovskites.[1–3] The usage of $SnCl_2$ and H_3PO_2 as coordinating and reducing agents, respectively, is important to limit the oxidation of the tin (II) perovskites in aqueous solution.[4–6] The solid precursors (tin chloride and A-site cation salts, but not CsI) were added to vials with an appropriate amount of 1:1 v/v HI and H_3PO_2 solution. For each of the 2D tin perovskites, the liquid 4FPEA was added dropwise to this solution prior to heating, which provided an initial black precipitate. Then each solution was heated to full dissolution at 120 °C. Importantly for $(4FPEA)_2(Cs)Sn_2I_7$, the CsI was added to this hot solution with all other precursors already dissolved in order to produce the $n = 2$ phase without co-crystallizing yellow needle impurities (yellow-phase δ-$CsSnI_3$ and recrystallized CsI). After all solids were fully dissolved, the solution was slow cooled to room temperature to yield large black single crystals with plate morphology. Note that the optical bandgaps are sufficiently narrow such that all $(4FPEA)_2(A)_{n-1}Sn_nI_{3n+1}$ phases appear black to the naked eye, unlike the distinct colors observed in lead-based 2D lead iodide phases. Thus, the phase purity of the samples was confirmed through PXRD and PL analyses.

X-ray diffraction. Powder X-ray diffraction (PXRD) measurements were taken on crushed single crystals using a Bruker D8 Advance X-ray diffractometer with Cu Kα radiation. For the air stability measurements, the tin perovskite powders were stored in ambient air but dark conditions to limit photodegradation, and taken out periodically for the PXRD measurement and returned shortly afterward. The lab relative humidity (RH) was tracked with a digital hygrometer, and was in the range of RH = 20-45% during the four-week period. Single-crystal X-ray diffraction (SCXRD) structure determination was performed on the five new quasi-2D perovskites reported herein: $(4FPEA)_2(Cs)Sn_2I_7$, $(4FPEA)_2(MA)Sn_2I_7$, $(4FPEA)_2(FA)Sn_2I_7$, $(4FPEA)_2(MA)_2Sn_3I_{10}$,

and also $(PEA)_2(FA)Sn_2I_7$. SCXRD data collection was performed using a Bruker Quazar SMART APEXII diffractometer with Mo K_α (λ = 0.71073 Å) radiation for most new phases (operation at 50 kV and 0.6 mA), or a Bruker D8 VENTURE PhotonIII four-circle diffractometer with Cu Kα (λ = 1.54178 Å) radiation for the $(4FPEA)_2(Cs)Sn_2I_7$ phase (operation at 50 kV and 1.1 mA). Each structure was solved by intrinsic phasing and refined using least-squares cycles and difference Fourier maps to locate remaining non-hydrogen atoms.[7,8] All non-hydrogen atoms were refined with anisotropic displacement coefficients. All hydrogen atoms were included in the structure factor calculation at idealized positions and were allowed to ride on the neighboring atoms with relative isotropic displacement coefficients. The $(4FPEA)_2(MA)Sn_2I_7$ and $(4FPEA)_2(FA)Sn_2I_7$ crystals chosen for the SCXRD diffraction experiments proved to be non-merohedral twins with 43.21% and 28.07% second component contributions, respectively. Reflections from only the first twin domains were included in the data refinement for these compounds. The refinement details for each of the 4FPEA-based phases are provided in the main text Table 1, and the refinement details for the $(PEA)_2(FA)Sn_2I_7$ phase are provided in Table S2.

Structural distortion measurements and calculations. The various bond lengths, bond angles, dihedral angles, etc. were directly measured for each crystal structure using OLEX2 and VESTA software.[9,10] Structural distortion parameters such as σ^2 and D were calculated from the measured bond angles and bond lengths. Averages of structural parameters are reported for cases where multiple crystallographically unique octahedra were present.

Inductively coupled plasma-optical emission spectroscopy (ICP-OES). ICP-OES measurements were conducted on an Agilent 5110 VDV instrument. Cs and Sn standard solutions were prepared by dissolving either 47.9 mg CsI (950 ppm CsI or ~500 ppm Cs) or 48.9 mg $SnCl_2\cdot 2H_2O$ (977.5 ppm salt or ~500 ppm Sn) in 50 mL of 1% HNO_3 solution. These standard solutions were then serially diluted with additional 1% HNO_3 to obtain standards with concentration ranges between 1-500 ppm. The $(4FPEA)_2(Cs_{0.57}MA_{0.43})Sn_2I_7$ sample was prepared by dissolving 12.7 mg of crystals in 50 mL of 1% HNO_3. The final Cs:MA ratio was calculated from the Cs:Sn ratio from the elemental analysis and the known 1:2 ratio of A-cation to metal for the n = 2 $(4FPEA)_2(A)Sn_2I_7$ phase.

Proton (^1H) NMR. Approximately 10.0 mg of the $(4FPEA)_2(MA/FA)Sn_2I_7$ alloyed crystals were dissolved in 1.0 mL D_2O. From this solution, 600 μL were pipetted into NMR sample tube for ^1H NMR analysis using a Bruker Avance 400 MHz spectrometer with a BBFO probe.

Photoluminescence. Photoluminescence (PL) measurements were taken on single crystals of each 2D tin perovskite using a Horiba LABRAM HR Evolution Raman/PL Spectrometer under 532 nm excitation.

Time-resolved photoluminescence. TRPL measurements were collected on exfoliated perovskite crystals using a custom-build hyperspectral microscope. The sample was excited with a 370 nm pulsed laser diode (Horiba DeltaDiode DD375L, 10 MHz rep. rate, 50 ps pulse duration). The beam was condensed through a 10x microscope objective (Olympus UMPlanF1, NA 0.30) to make a focused spot on the sample. Photoluminescence (PL) was collected through the same objective and the excitation light was filtered twice through a dichroic mirror and a 450 nm longpass filter (ThorLabs FELH0450, OD5+). The PL spot was imaged through a tube lens and passed through a slit into an imaging spectrograph (Princeton Instruments Acton sp2300i) where it was dispersed onto an avalanche photodiode (APD, IDQ ID-100-20-ULN). The grating was positioned such that the maximum emission wavelength for each phase was relayed onto the APD. TRPL transients were generated using a time-correlated single photon counting module (Becker and Hickl SPC-130). The TRPL spectra were then fit using a biexponential decay function, $I = A_1 e^{-t/\tau_1} + A_2 e^{-t/\tau_2} + y_0$, where τ_2 is taken as the TRPL lifetime. Several objects of each composition were surveyed to obtain a representative average for each composition.

Lasing measurements. Lasing measurements were performed using a femtosecond laser (1030 nm, pulse width ≈ 300 fs, Pharos, Light Conversion) with an external harmonic generator (Hiro, Light Conversion) to generate a second harmonic 515 nm pump. The laser beam was then collimated into a fiber collimator and sent to a Nikon LV100ND upright microscope focused by a 100x objective onto the exfoliated microflakes of 2D tin perovskites that were placed in a cryostat (THMS60, Linkam) and cooled down to 77 K. An Ocean Optics Maya2000 Pro spectrometer collected the PL signals. The 2D tin perovskite crystals were exfoliated in an N_2 filled glovebox and then the exfoliated microflakes were transferred to the cryostat, ensuring no exposure to ambient air.

Supporting Tables

Table S1. Summary of the precursor recipes for synthesizing the $(4FPEA)_2(A)_{n-1}Sn_nI_{3n+1}$ (n = 1-3, A = Cs^+, MA, FA) perovskites studied herein.

Compound	LA (mmol)	AX (mmol)	$SnCl_2 \cdot 2H_2O$ (mmol)	HI (mL)	H_3PO_2 (mL)
$(4FPEA)_2SnI_4$	0.40 4FPEA	-	0.20	1.5	1.5
$(PEA)_2(FA)Sn_2I_7$	0.10 PEAI	0.6 FAI	0.20	1.0	0.5
$(4FPEA)_2(Cs)Sn_2I_7$	0.75 4FPEA	0.5 CsI*	1.0	2.5	2.5
$(4FPEA)_2(MA)Sn_2I_7$	0.75 4FPEA	1.5 MAI	1.0	2.0	2.0
$(4FPEA)_2(FA)Sn_2I_7$	0.75 4FPEA	1.5 FAI	1.0	2.0	2.0
$(4FPEA)_2(Cs_{0.57}MA_{0.43})Sn_2I_7$	0.75 4FPEA	0.25 CsI*<br>0.75 MAI	1.0	2.25	2.25
$(4FPEA)_2(MA_{0.45}FA_{0.55})Sn_2I_7$	0.75 4FPEA	0.75 MAI<br>0.75 FAI	1.0	2.0	2.0
$(4FPEA)_2(MA)_2Sn_3I_{10}$	0.375 4FPEA	2.3 MAI	0.5	0.5**	0.5

* CsI powder added into hot solution (120 °C) after all other precursors dissolved.
** Organic co-solvent (1.0 mL acetonitrile) was also introduced to improve the phase purity.

Table S2. Crystal data and structure refinement for $(PEA)_2(FA)Sn_2I_7$.

Empirical formula	$[C_8H_{12}N]_2[CH_5N_2]Sn_2I_7$
Formula weight	1415.12
Temperature/K	100.01
Crystal system	Triclinic
Space group	*P*1
a/Å	8.726(3)
b/Å	8.728(3)
c/Å	22.227(6)
α/°	93.640(5)
β/°	97.547(19)
γ/°	90.298(7)
Volume/Å^3	1674.6(9)
Z	2
ρ_{calc}g/cm^3	2.806
μ/mm^{-1}	7.957
F(000)	1260.0
Crystal size/mm^3	0.05 × 0.035 × 0.005
Radiation	Mo Kα (λ = 0.71073)
2Θ range for data collection/°	3.704 to 61.044
Index ranges	$-12 \le h \le 12$, $-12 \le k \le 12$, $-31 \le l \le 31$
Reflections collected	41352
Independent reflections	19255 [R_{int} = 0.0354, R_{sigma} = 0.0578]
Data/restraints/parameters	19255/334/543
Goodness-of-fit on F^2	1.028
Final R indexes [I>=2σ (I)]	R_1 = 0.0390, wR_2 = 0.0717
Final R indexes [all data]	R_1 = 0.0582, wR_2 = 0.0781
Largest diff. peak/hole / e Å^{-3}	1.65/-1.55
Flack parameter	0.07(3)

Table S3. TRPL lifetimes of the $(4FPEA)_2(A)_{n-1}Sn_nI_{3n+1}$ (n = 1-3, A = Cs^+, MA, FA) perovskites, estimated from biexponential decay fitting of spectra shown in Figures S3-6.

Compound	Obj. 1 TRPL (ns)	Obj. 2 TRPL (ns)	Obj. 3 TRPL (ns)	Obj. 4 TRPL (ns)	Obj. 5 TRPL (ns)	Ave. TRPL (ns)
$(4FPEA)_2SnI_4$	1.36	1.31	0.81	1.3	-	1.2 ± 0.3
$(4FPEA)_2(Cs)Sn_2I_7$	1.31	1.03	2.19	0.87	1.35	1.4 ± 0.5
$(4FPEA)_2(MA)Sn_2I_7$	5.22	18.47	5.14	3.08	22.97	11.0 ± 9.1
$(4FPEA)_2(FA)Sn_2I_7$	1.38	0.87	5.77	1.64	9.99	3.9 ± 3.9

Table S4. ICP-OES analysis of $(4FPEA)_2(Cs,MA)Sn_2I_7$ with Cs and Sn standards.

	Cs (697.3 nm, radial)			Sn (235.5 nm, radial)	
**Sample**	**ppm**	Intensity (a.u.)	**Sample**	**ppm**	Intensity (a.u.)
500cal	490.49	3368.7	500cal	500	50013.31
250cal	255.96	1754.2	250cal	250	24759.13
125cal	124.65	850.23	125cal	125	12433.77
100cal	92.15	626.47	100cal	100	9512.32
50cal	48.65	327.03	50cal	50	4656.87
n=2 perovskite	***12.74129***	79.83	n=2 perovskite	***39.91715***	3636.88
	= 0.637 mg Cs = ***4.79 mmol Cs***			= 1.996 mg Sn = ***16.81 mmol Sn***	

Table S5. Summary of the structural distortion parameters and cage classifications for reported quasi-2D n = 2 lead and tin iodide perovskites.

Compound	CCDC No.	Reference	Temperature (K)	Ave. B-I-B IP angle (°)	Ave. B-I-B OOP angle (°)	σ_2^2 (deg^2)	Ave. OOP I-Sn-Sn-I angle (°)	Cage Type
$(BA)_2(MA)Sn_2I_7$	2240839	[11]	285	169.25	167.69	15.39	12.62	Tilted
$(BA)_2(FA)Sn_2I_7$	2190110	[12]	293	171.13	172.70	28.19	12.27	Tilted
$(BA)_2(DMA)Sn_2I_7$	2190111	[12]	293	167.03	179.50	44.94	13.13	Buckled *
$(BA)_2(GA)Sn_2I_7$	2190113	[12]	293	168.54	171.91	14.99	13.45	Tilted
$(BA)_2(AA)Sn_2I_7$	2190112	[12]	293	170.25	174.88	20.68	12.04	Tilted
$(3,5FBZ)_2(FA)Sn_2I_7$	2171384	[13]	294	167.40	170.11	15.15	13.35	Tilted
$(BA)_2(MA)Pb_2I_7$	1478376	[1]	293	167.24	165.63	11.13	14.04	Tilted
$(BA)_2(FA)Pb_2I_7$	2011909	[14]	293	169.40	171.29	26.51	13.72	Tilted
$(BA)_2(DMA)Pb_2I_7$	2011907	[14]	293	166.76	178.49	49.75	13.67	Buckled *
$(BA)_2(GA)Pb_2I_7$	2011908	[14]	293	168.15	171.72	19.31	14.33	Tilted
$(PA)_2(MA)Pb_2I_7$	1904719	[15]	293	166.54	163.99	16.96	15.58	Tilted
$(PA)_2(FA)Pb_2I_7$	1888557	[16]	260	168.64	170.68	36.63	15.06	Tilted
$(PA)_2(DMA)Pb_2I_7$	2013884	[17]	200	163.83	180.00	91.32	15.80	Buckled **
$(PA)_2(DMA)Pb_2I_7$	2268601	[18]	100	166.15	167.11	82.17	16.06	Buckled **
$(PA)_2(EA)Pb_2I_7$	2268602	[18]	200	161.71	174.61	49.05	12.03	Tilted
$(PA)_2(GA)Pb_2I_7$	1891567	[19]	298	167.61	171.95	28.86	15.26	Tilted
$(PA)_2(AA)Pb_2I_7$	2083467	[20]	293	168.01	173.77	28.65	14.94	Tilted**
$(PA)_2(AA)Pb_2I_7$	2268600	[18]	100	162.27	162.45	60.82	16.47	Tilted **
$(HA)_2(MA)Pb_2I_7$	1904716	[15]	293	160.23	176.60	27.30	9.31	Tilted [b]
$(HA)_2(FA)Pb_2I_7$	1842373	[21]	173	160.37	176.845	56.26	10.09	Buckled *,[b]
$(HA)_2(GA)Pb_2I_7$	1888368	[22]	100	160.22	178.87	56.27	10.09	Buckled *

$(OA)_2(MA)Pb_2I_7$	2072075	[23]	293	162.40	174.12	26.38	10.16	Tilted
$(IEA)_2(MA)Pb_2I_7$	2223698	[24]	293	161.39	171.85	36.55	13.02	Tilted
$(IEA)_2(FA)Pb_2I_7$	2306159	[25]	223	159.15	173.46	14.03	9.70	Tilted
$(IPA)_2(FA)Pb_2I_7$	2077148	[26]	299.5	161.69	175.48	33.29	9.87	Tilted
$(IBA)_2(FA)Pb_2I_7$	2320484	[25]	293	162.35	173.53	28.96	12.45	Tilted
$(c\text{-}C4A)_2(MA)Pb_2I_7$	2405918	[27]	100	168.95	162.17	11.47	13.17	Tilted
$(BZA)_2(MA)Pb_2I_7$	1876243	[28]	300	159.92	175.30	29.62	10.63	Tilted
$(4FBZ)_2(MA)Pb_2I_7$	1819860	[29]	296	159.77	175.54	24.04	10.28	Tilted
$(4BrBZ)_2(MA)Pb_2I_7$	2234100	[30]	298	160.05	174.60	22.36	10.33	Tilted
$(iPA)_2(iPA)Sn_2I_7$	1530309	[31]	293	171.42	178.1	44.51	12.91	Buckled
$(iPA)_2(MA)Pb_2I_7$	N/A	[32]	298	168.30	163.08	26.32	13.90	Tilted
$(iBA)_2(MA)Pb_2I_7$	1876241	[28]	300	159.54	176.28	20.91	8.89	Tilted [a]
$(iBA)_2(GA)Pb_2I_7$	2078064	[33]	250	163.69	177.50	29.88	11.65	Tilted [a]
$(iPeA)_2(GA)Pb_2I_7$	2353249	[34]	320	173.15	175.66	14.70	8.64	Tilted [a]
$(S\text{-}\beta\text{-}MPEA)_2(MA)Pb_2I_7$	1936623	[35]	296	152.61	177.73	27.93	4.02	Tilted [a]
$(PEA)_2(MA)Sn_2I_7$	2240837	[11]	150	158.15	180.00	10.99	2.31	Balanced [b]
$(4FPEA)_2(Cs)Sn_2I_7$	2457342	This work	294	157.65	180.00	18.44	4.30	Balanced
$(4FPEA)_2(MA)Sn_2I_7$	2457339	This work	295	159.60	179.40	12.66	4.70	Balanced
$(4FPEA)_2(FA)Sn_2I_7$	2457338	This work	295	160.65	180.00	21.96	4.89	Balanced
$(3T)_2(MA)Sn_2I_7$	2376615	[36]	150	156.70	178.55	14.06	1.82	Balanced
$(FSA)_2(MA)Sn_2I_7$	1887147	[37]	297	159.28	179.11	5.66	5.66	Balanced
$(4MeBZ)_2(MA)Pb_2I_7$	141191	[38]	298	157.12	178.21	28.36	8.07	Balanced
$(PEA)_2(MA)Pb_2I_7$	1978042	[39]	100	153.87	179.93	16.73	1.89	Balanced [b]
$(4FPEA)_2(MA)Pb_2I_7$	1483085	[40]	100	153.32	179.96	20.85	4.39	Balanced
$(4PhPEA)_2(MA)Pb_2I_7$	1940830	[41]	100	154.45	179.24	20.35	4.18	Balanced
$(2T)_2(MA)Pb_2I_7$	2151387	[42]	150	153.50	179.26	29.70	2.38	Balanced

$(3T)_2(MA)Pb_2I_7$	2151390	[42]	150	152.71	179.50	27.36	2.24	Balanced
$(4Tm)_2(MA)Pb_2I_7$	2151391	[42]	150	154.47	179.24	25.74	0.94	Balanced
$(HOOCPrA)_2(MA)Pb_2I_7$	267397	[43]	293	159.98	179.94	23.05	5.21	Balanced
$(CMA)_2(MA)Pb_2I_7$	N/A	[44]	296	157.57	178.75	28.15	7.01	Balanced [b]
$(ThMA)_2(MA)Pb_2I_7$	1976431	[45]	293	156.26	178.55	22.35	7.30	Balanced [b]
$(2IPrA)_2(MA)Pb_2I_7$	2141818	[46]	293	156.37	178.91	12.34	6.56	Balanced [b]
$(PEA)_2(FA)Sn_2I_7$	2457341	This work	100	158.41	177.70	35.57	4.91	Buckled [b,c]
$(ThMA)_2(FA)Pb_2I_7$	1974807	[47]	150	156.11	175.95	58.88	9.54	Buckled [b]
$(ThMA)_2(FA)Pb_2I_7$	1976429	[45]	296	156.47	176.90	37.35	9.38	Buckled [b,c]
$(2IPrA)_2(FA)Pb_2I_7$	2219451	[48]	150	157.74	174.30	58.46	11.27	Buckled [b]
$(2FBZ)_2(MA)Pb_2I_7$	2189432	[49]	100	156.49	177.66	38.49	8.78	Buckled [c]
$(BrFBZ)_2(MA)Sn_2I_7$	2286644	[50]	100	158.85	177.14	54.38	9.94	Buckled
$(BrFBZ)_2(FA)Sn_2I_7$	2286641	[50]	100	160.23	174.90	88.59	11.43	Buckled
$(BrFBZ)_2(MA)Pb_2I_7$	2286646	[50]	100	156.03	177.19	47.78	9.72	Buckled
$(BrFBZ)_2(FA)Pb_2I_7$	2286645	[50]	100	156.84	174.67	111.19	12.28	Buckled
$(CMA)_2(FA)Pb_2I_7$	2320590	[51]	295	160.17	178.16	50.46	8.45	Buckled [b]
$(AlA)_2(MA)Pb_2I_7$	2141812	[46]	293	163.97	177.30	91.87	0.25	Buckled
$(2FBZ)_2(FA)Pb_2I_7$	2189433	[49]	100	157.32	174.53	86.30	7.49	Buckled
$(R\text{-}4BrMBA)_2(FA)Pb_2I_7$	2193330	[52]	150	155.49	175.46	60.39	8.54	Buckled
$(S\text{-}4BrMBA)_2(FA)Pb_2I_7$	2193331	[52]	250	156.84	177.57	52.39	8.46	Buckled

* Structure exhibits different cage types compared to analogues with same spacer cation due to oversized A-cation. ** The centrosymmetric crystal structures determined at higher T are used to determine the structural parameters. Phase transition and/or oversized A-cation can give greater structural distortion under low T.

[a] Structure exhibits only minor tilting and is on the cusp of becoming balanced.

[b] Structure is on the threshold of balanced and buckled regimes. The choice of A-cation (MA vs FA) pushes the cage class towards one regime or another.

[c] Structure exhibits octahedra with only minor metal off-centering and is on the cusp of becoming balanced.

Supporting Figures

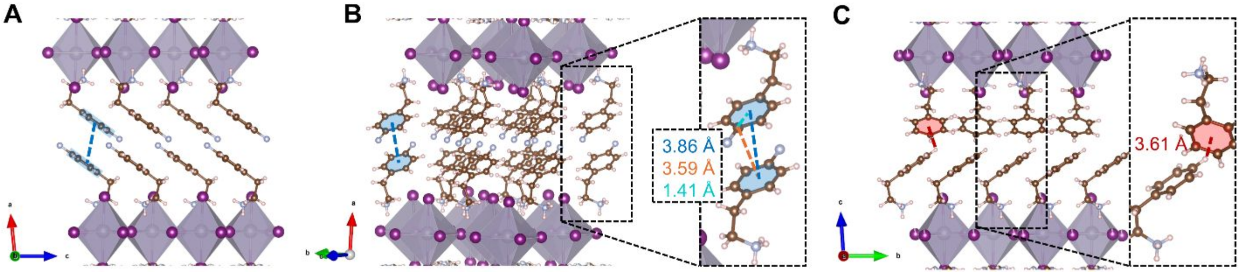

Figure S1. Offset face-to-face π-stacking of 4FPEA spacer cations in $(4FPEA)_2(FA)Sn_2I_7$ as shown along the (A) [010] direction and (B) [0$\bar{1}$1] direction, with inset showing distances for the ring centroid-to-centroid, centroid-to-plane, and offset. (C) The edge-to-face π-stacking of PEA spacer cations in $(PEA)_2(FA)Sn_2I_7$ along the [100] direction is shown for comparison. The crystal structure of $(PEA)_2(FA)Sn_2I_7$ was solved in this work (details shown in Table S2) and the minor disorder components are omitted for clarity.

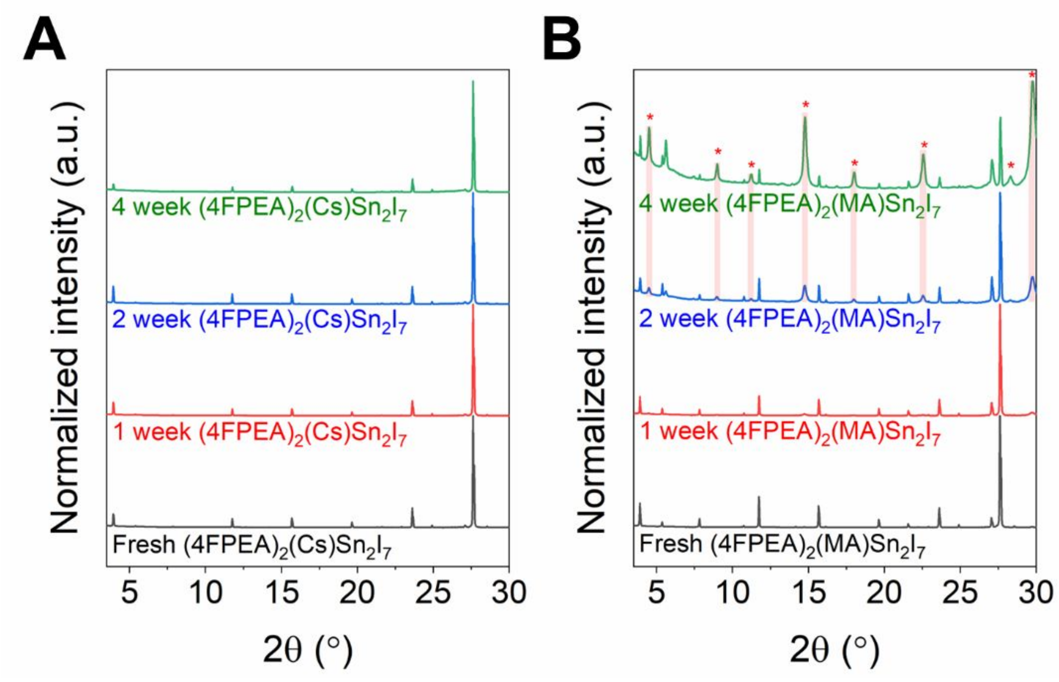

Figure S2. Powder X-ray diffraction patterns for (A) $(4FPEA)_2(Cs)Sn_2I_7$ and (B) $(4FPEA)_2(MA)Sn_2I_7$ as crushed crystals were aged under ambient air with RH = 20-45%. Red highlights and asterisks indicate the growth of non-(h00) reflections corresponding to degradation products. These PXRD patterns supplement the air stability assessment of the other $(4FPEA)_2(A)_{n-1}Sn_nI_{3n+1}$ (n = 1, 2, A = FA) phases shown in Figure 4 of the main text.

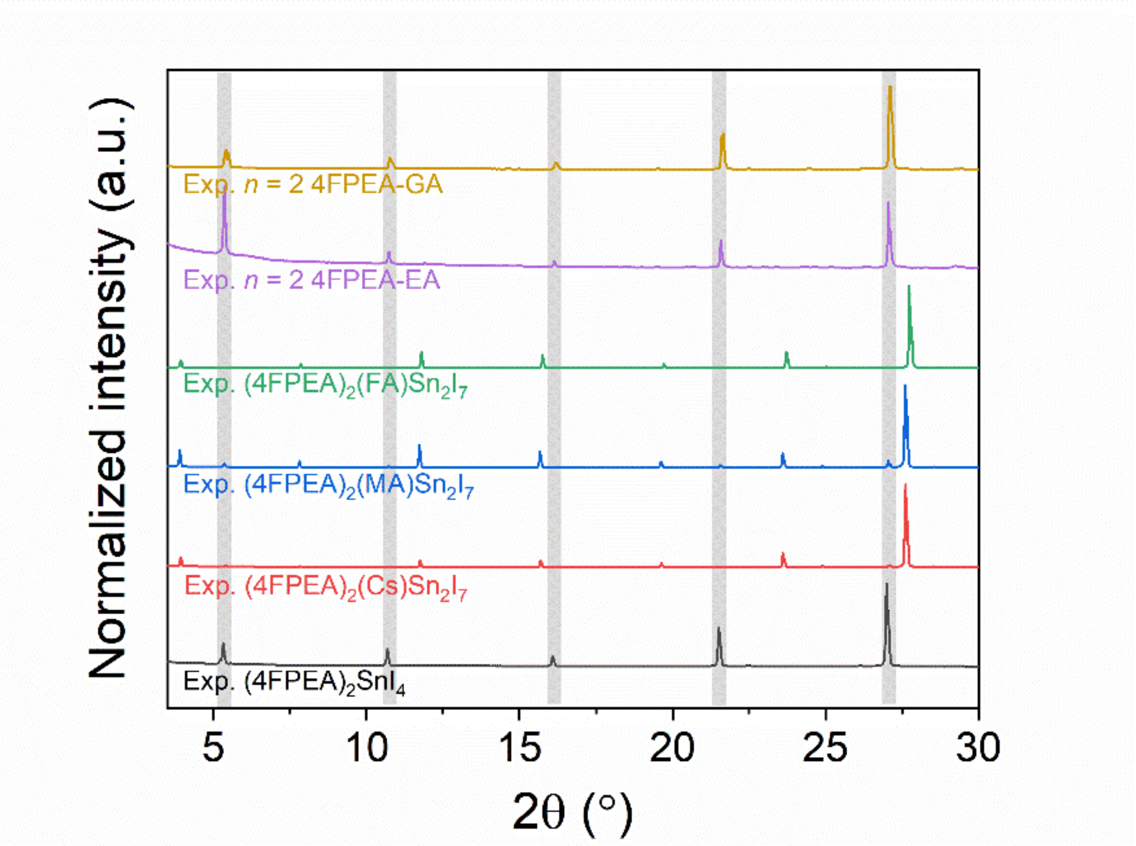

Figure S3. Experimental powder X-ray diffraction patterns for n = 1 $(4FPEA)_2SnI_4$ and n = 2 $(4FPEA)_2(A)Sn_2I_7$ perovskites with various A-cations. Attempts to synthesize the analogous EA and GA phases using similar precursor stoichiometry to the working n = 2 $(4FPEA)_2(FA)Sn_2I_7$ recipe yielded only n = 1 crystals (black highlights correspond to the (h00)-type reflections from the n = 1 phase).

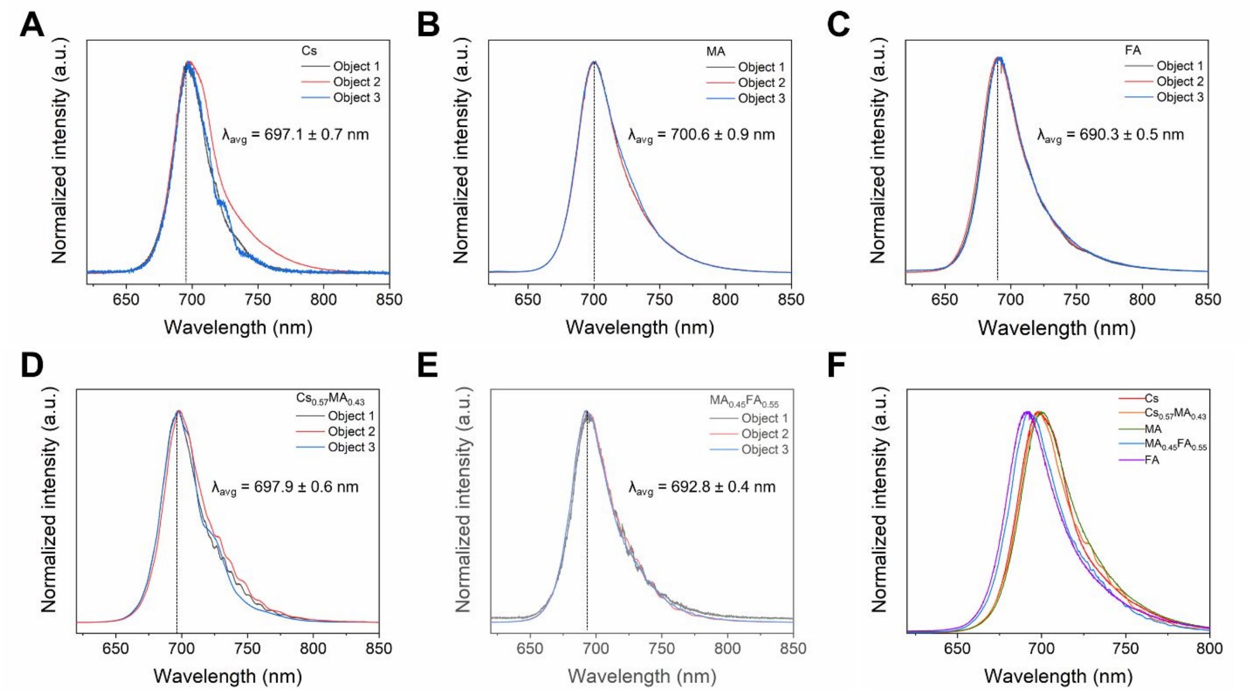

Figure S4. Photoluminescence (PL) spectra for (A) $(4FPEA)_2(Cs)Sn_2I_7$, (B) $(4FPEA)_2(MA)Sn_2I_7$, (C) $(4FPEA)_2(FA)Sn_2I_7$, (D) $(4FPEA)_2(Cs_{0.57}MA_{0.43})Sn_2I_7$ and (E) $(4FPEA)_2(MA_{0.45}FA_{0.55})Sn_2I_7$ across multiple objects. (F) Comparison of representative PL spectra for the different $(4FPEA)_2(A)Sn_2I_7$ end phases and A-cation alloys.

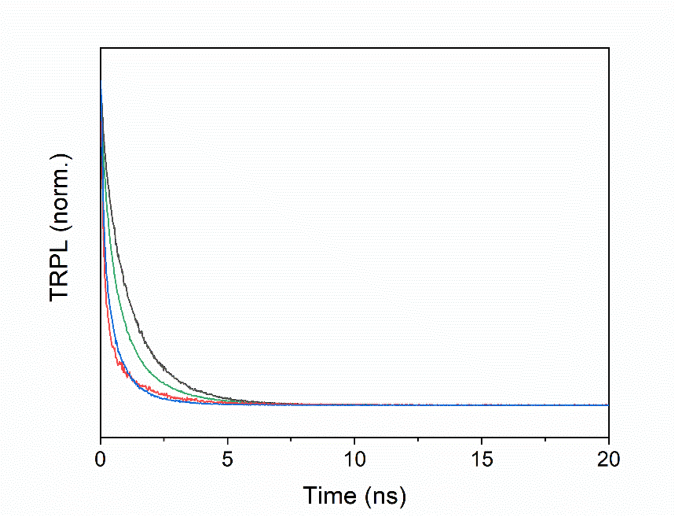

Figure S5. Time-resolved photoluminescence (TRPL) spectra of different exfoliated flakes of $(4FPEA)_2SnI_4$.

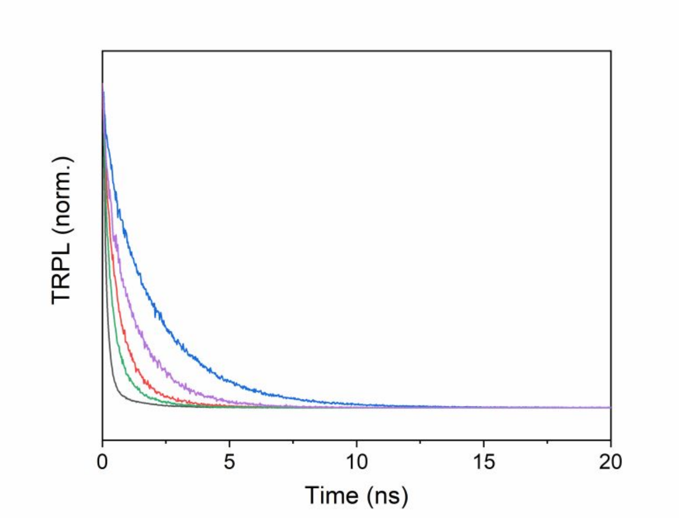

Figure S6. Time-resolved photoluminescence (TRPL) spectra of different exfoliated flakes of $(4FPEA)_2(Cs)Sn_2I_7$.

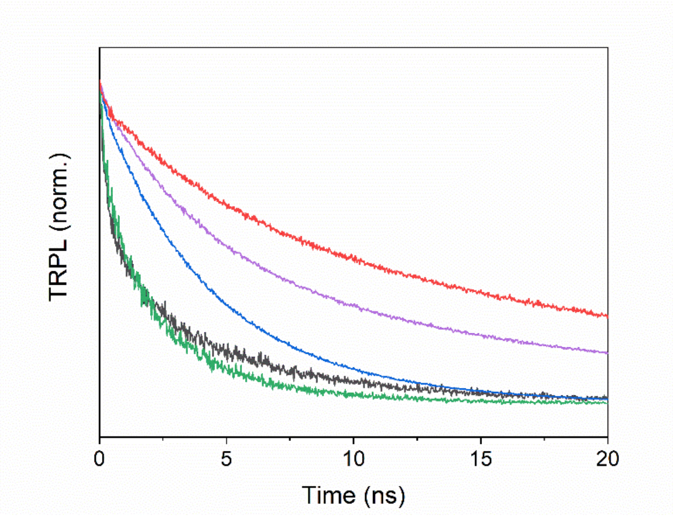

Figure S7. Time-resolved photoluminescence (TRPL) spectra of different exfoliated flakes of $(4FPEA)_2(MA)Sn_2I_7$.

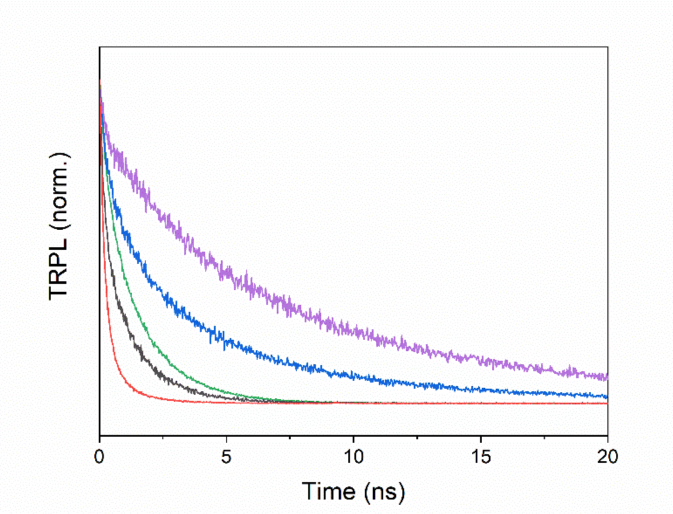

Figure S8. Time-resolved photoluminescence (TRPL) spectra of different exfoliated flakes of $(4FPEA)_2(FA)Sn_2I_7$.

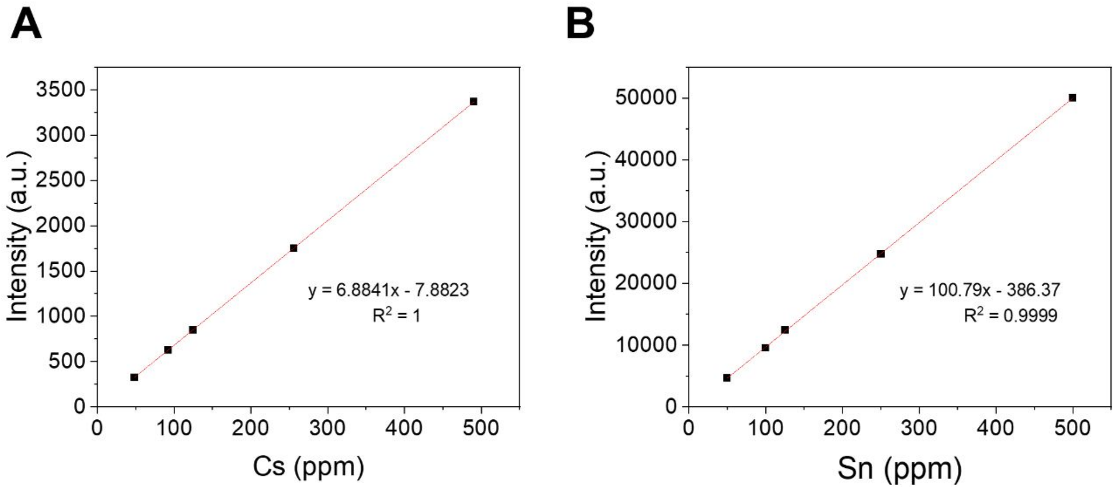

Figure S9. ICP-OES calibration curves for (A) Cs and (B) Sn standards.

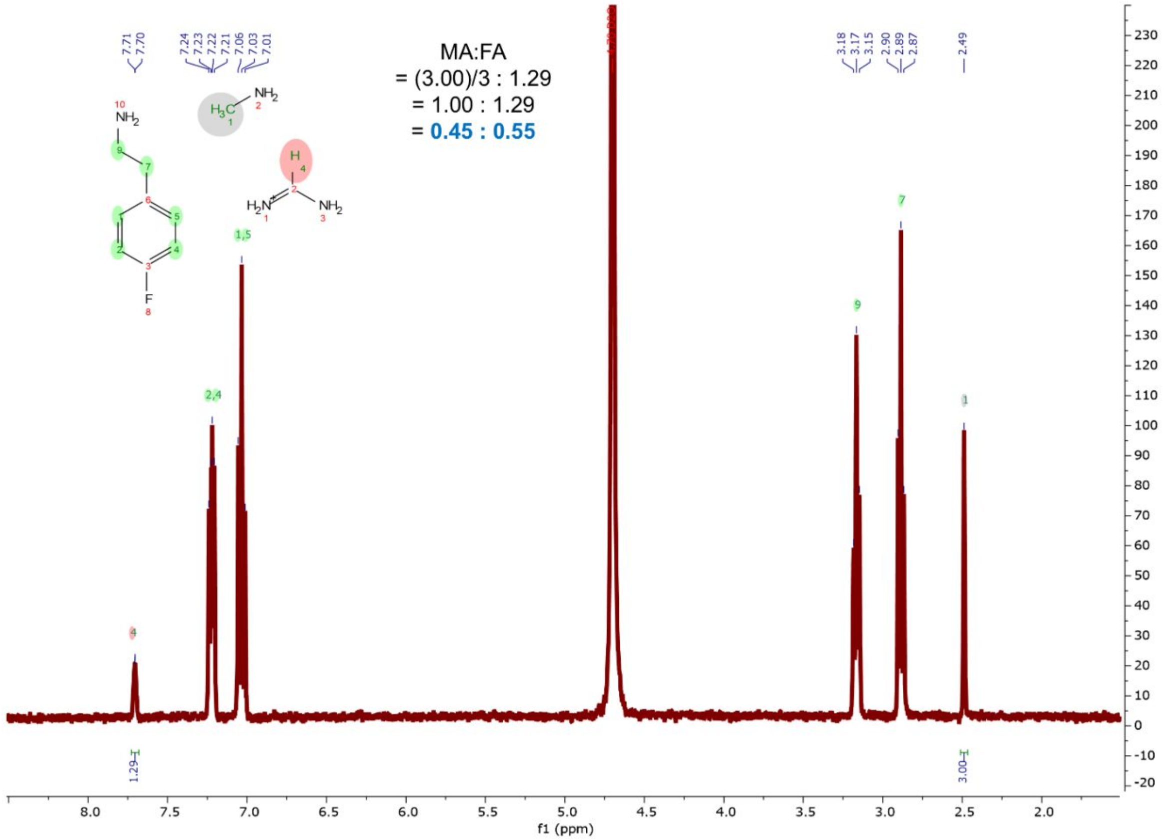

Figure S10. ^{1}H NMR spectrum and integration for $(4FPEA)_2(MA_{0.45}FA_{0.55})Sn_2I_7$ crystals dissolved in D_2O.

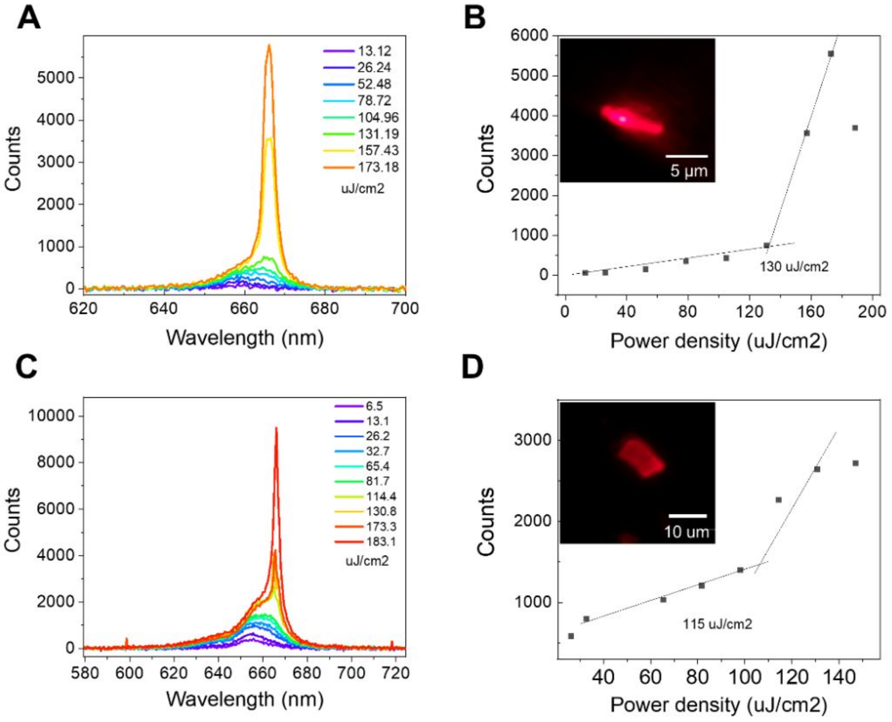

Figure S11. (A,C) Fluence dependent PL spectra and (B,D) pump fluence dependent PL intensity for different exfoliated microflakes of $(4FPEA)_2SnI_4$ at 77 K. The insets show the optical images of the microflakes under excitation.

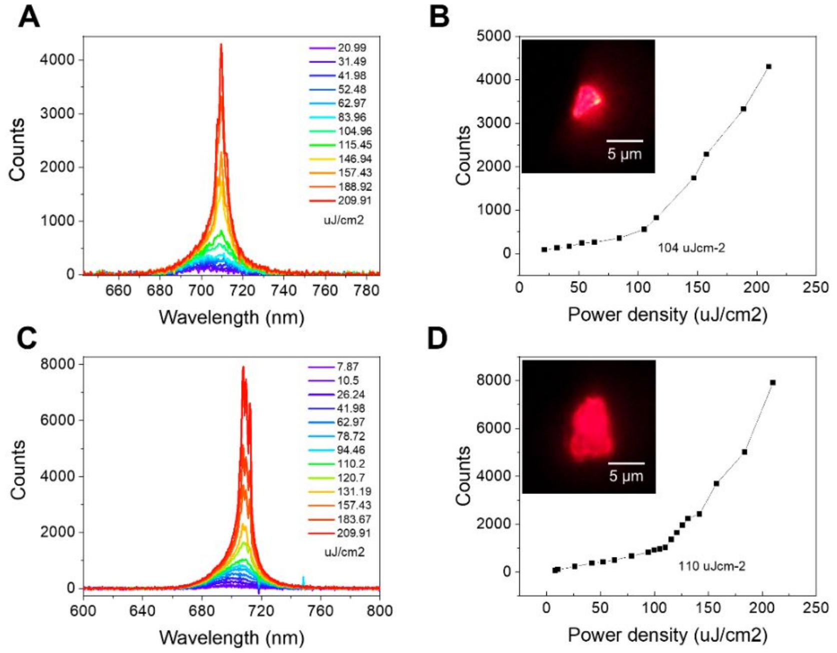

Figure S12. (A,C) Fluence dependent PL spectra and (B,D) pump fluence dependent PL intensity for different exfoliated microflakes of $(PEA)_2(FA)Sn_2I_7$ at 77 K. The insets show the optical images of the microflakes under excitation.

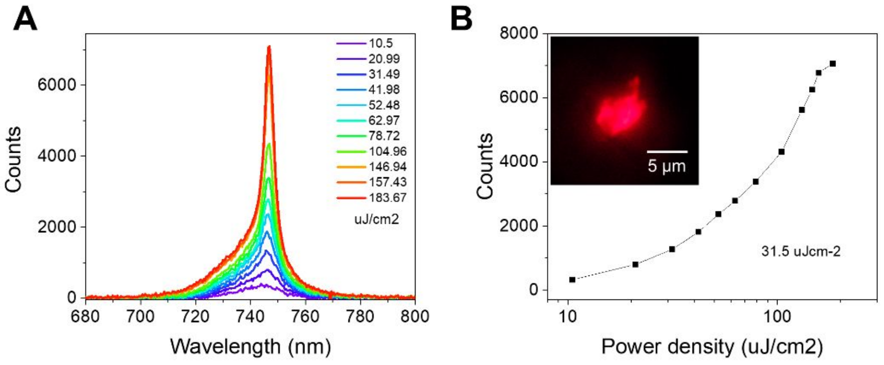

Figure S13. (A) Fluence dependent PL spectra and (B) pump fluence dependent PL intensity for an exfoliated microflake of $(4FPEA)_2(Cs)Sn_2I_7$ at 77 K. The inset shows the optical image of the microflake under excitation.

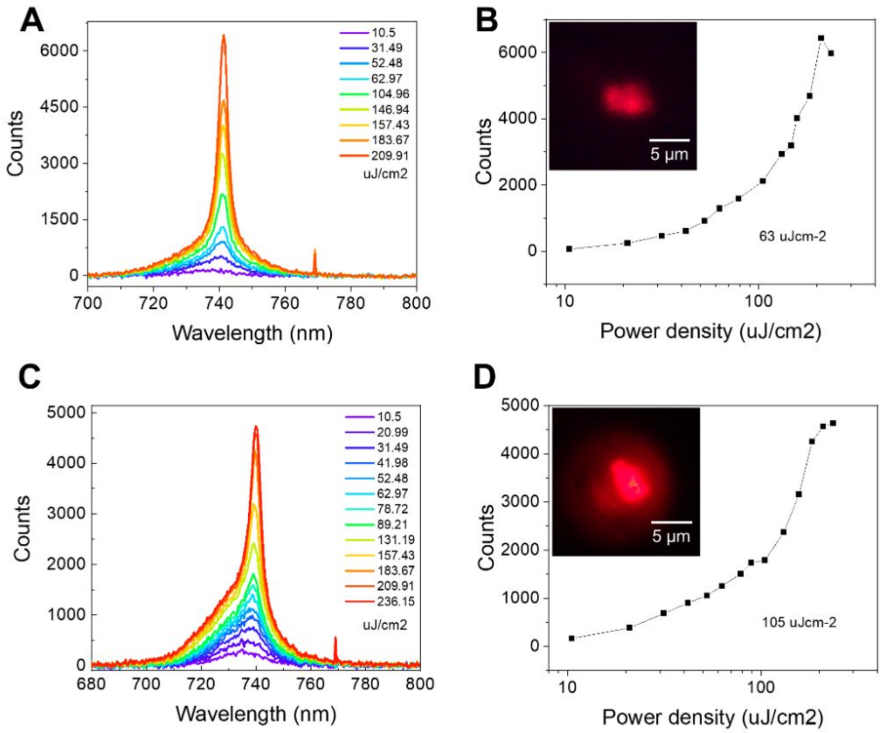

Figure S14. (A) Fluence dependent PL spectra and (B) pump fluence dependent PL intensity for two additional exfoliated microflakes of $(4FPEA)_2(MA)Sn_2I_7$ at 77 K. The insets show the optical images of the microflakes under excitation.

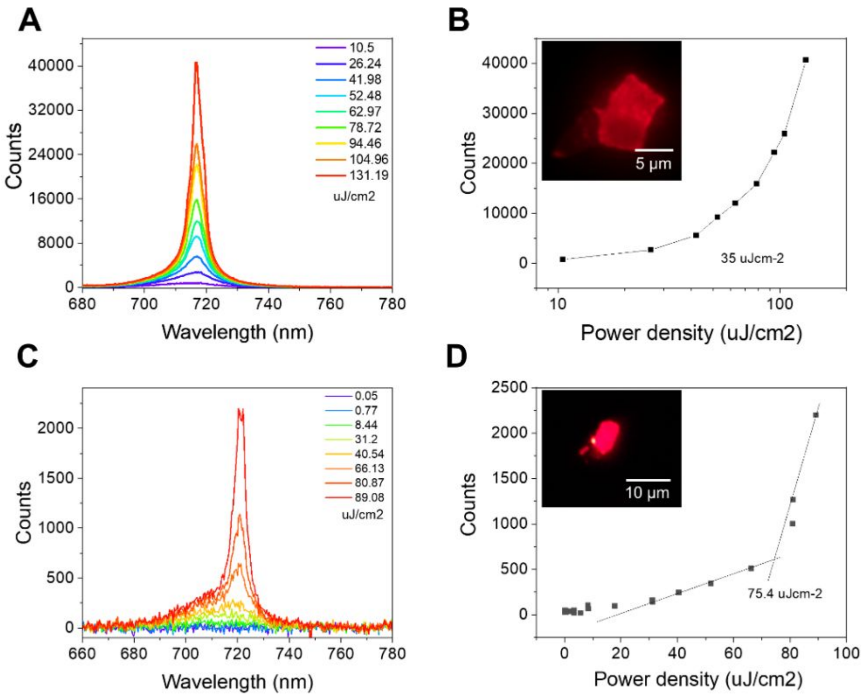

Figure S15. (A) Fluence dependent PL spectra and (B) pump fluence dependent PL intensity for different exfoliated microflakes of $(4FPEA)_2(FA)Sn_2I_7$ at 77 K. The insets show the optical images of the microflakes under excitation.

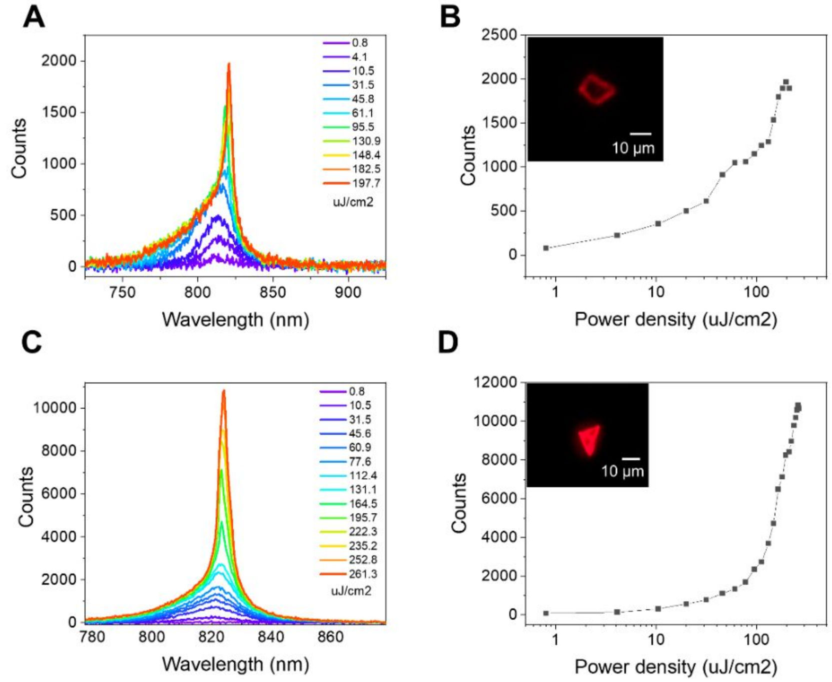

Figure S16. (A) Fluence dependent PL spectra and (B) pump fluence dependent PL intensity for different exfoliated microflakes of $(4FPEA)_2(MA)_2Sn_3I_{10}$ at 77 K. The insets show the optical images of the microflakes under excitation.

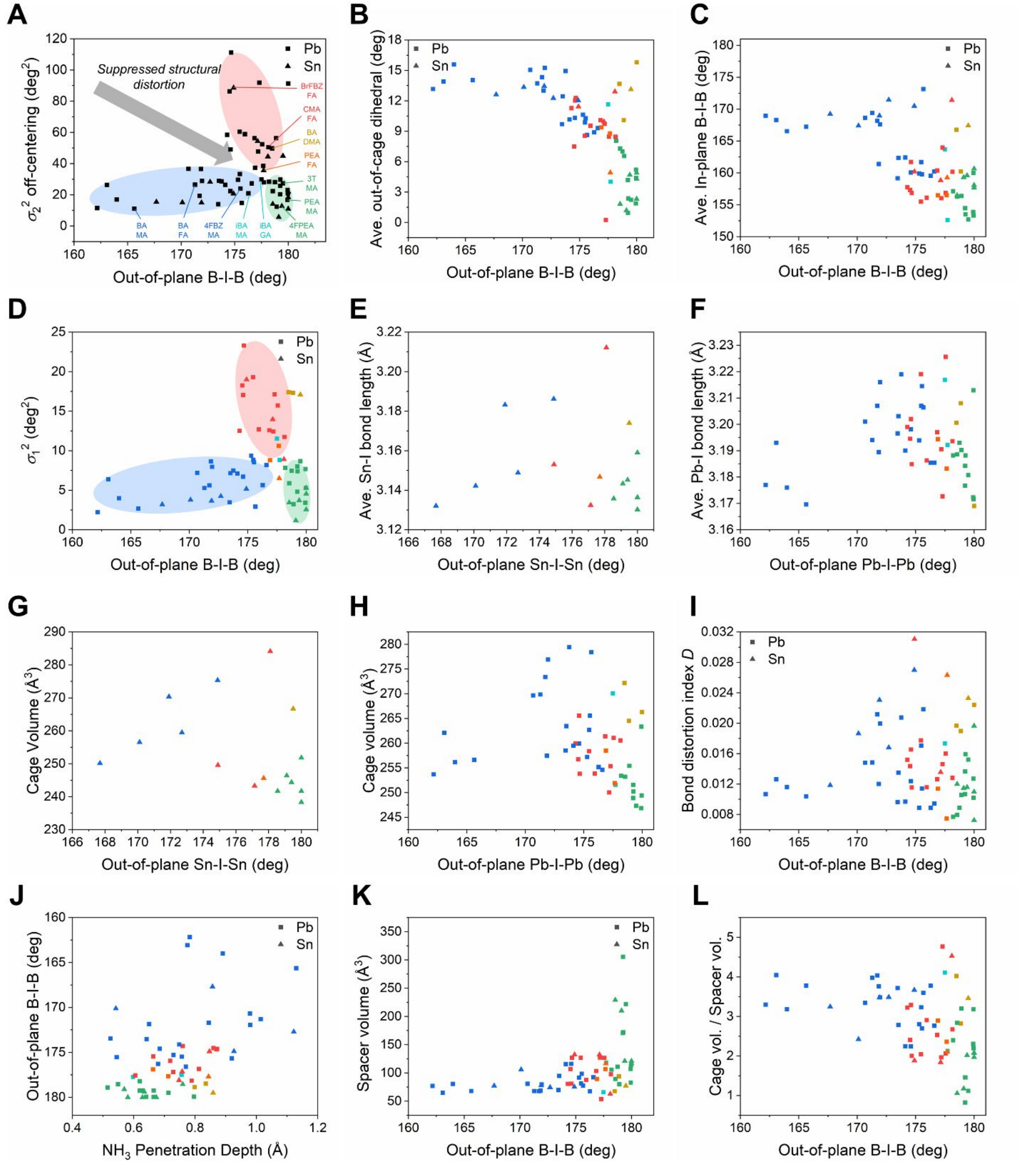

Figure S17. All 2D correlation plots of various structural parameters of the n = 2 lead and tin iodide RP perovskites surveyed. (A) Cage classification figure displayed in Figure 7B in the main text showing clear clustering behaviors. Weaker separations are observed in plots of correlating (B) in-plane B-I-B angles and (C) out-of-cage I-B-B-I dihedral angles vs. out-of-plane B-I-B angle. Similar correlations as panel A are found when (D) σ_1^2 bond-angle variance is plotted vs. out-of-plane B-I-B angle. However, poorer clustering behaviors can be seen in each case. No strong

correlations were found with average bond distances for (E) Sn-I distances and (F) Pb-I distances, however (G) Sn cage volumes and (H) Pb cage volumes are generally smallest for balanced cage types. Attempts to correlate other distortion parameters, such as (I) bond distortion index *D*, (J) NH_3^+ head group penetration depth (measured from the plane of terminal iodides), (K) spacer cation volume, and (L) cage to spacer volume ratio, with out-of-plane B-I-B angle, did not reveal clustering of data points. As visual guides, the colors of the data points displayed in panels (B-I) are based on their cage classes determined from panel A (blue = tilted, cyan = near-balanced minor tilted, green = balanced, orange = minor buckled, red = buckled, yellow = unusual case from oversized A-cation). All values are determined from their respective crystal structures listed in Table S5, and averages were used when multiple unique bonds, octahedra, etc. were present in the crystal structure.

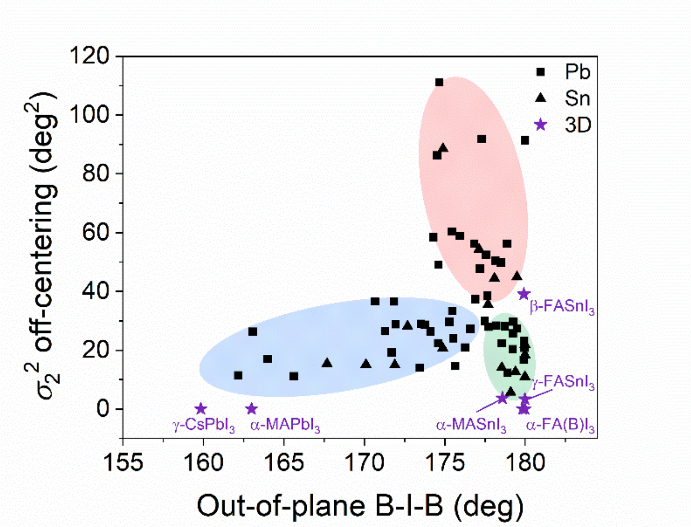

Figure S18. Cage classification plot based on correlation of distortion parameter with various known 3D ABI_3 phases added and denoted as "*". The analyzed structures include γ-$CsPbI_3$ (CCDC 1919371), α-$MAPbI_3$ (CCDC 967756), α-$MASnI_3$ (CCDC 968123), α-$FAPbI_3$ (CCDC 968126), α-$FASnI_3$ (CCDC), γ-$FASnI_3$ (CCDC 2004418), and β-$FASnI_3$ (CCDC 968120). Without the 3D ABI_3 phases, the plot is the same as one shown in Figure S17A and Figure 7B in the main text.

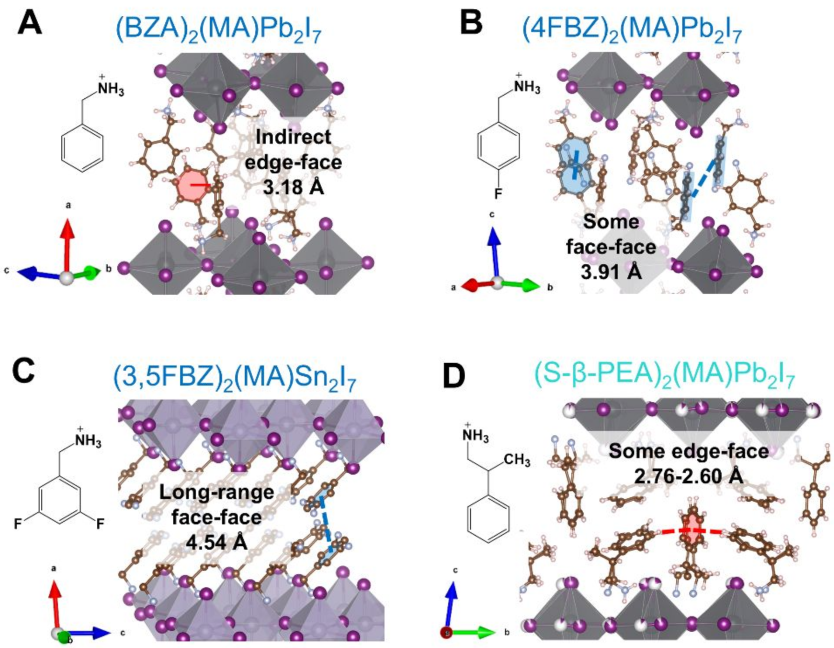

Figure S19. Crystal structures of reported $n = 2$ RP lead iodide perovskites with tilted cages due to poorer interlayer π-π interactions: (A) $(BZA)_2(MA)Pb_2I_7$,[28] (B) $(4FBZ)_2(MA)Pb_2I_7$,[29] (C) $(3,5FBZ)_2(MA)Sn_2I_7$,[13] (D) $(S\text{-}\beta\text{-}PEA)_2(MA)Pb_2I_7$.[35] The chemical structures of the corresponding aromatic spacer cations are displayed by the side. The interlayer π-π interactions are highlighted with dashed lines.

References cited in the Supporting Information

(1) Stoumpos, C. C.; Cao, D. H.; Clark, D. J.; Young, J.; Rondinelli, J. M.; Jang, J. I.; Hupp, J. T.; Kanatzidis, M. G. Ruddlesden-Popper Hybrid Lead Iodide Perovskite 2D Homologous Semiconductors. *Chem. Mater.* **2016**, *28* (8), 2852–2867. https://doi.org/10.1021/acs.chemmater.6b00847.

(2) Oswald, I. W. H.; Koegel, A. A.; Neilson, J. R. General Synthesis Principles for Ruddlesden-Popper Hybrid Perovskite Halides from a Dynamic Equilibrium. *Chem. Mater.* **2018**, *30* (23), 8606–8614. https://doi.org/10.1021/acs.chemmater.8b03817.

(3) Hou, J.; Li, W.; Zhang, H.; Sidhik, S.; Fletcher, J.; Metcalf, I.; Anantharaman, S. B.; Shuai, X.; Mishra, A.; Blancon, J.-C.; Katan, C.; Jariwala, D.; Even, J.; Kanatzidis, M. G.; Mohite, A. D. Synthesis of 2D Perovskite Crystals via Progressive Transformation of Quantum Well Thickness. *Nat. Synth.* **2024**, *3* (2), 265–275. https://doi.org/10.1038/s44160-023-00422-3.

(4) Pascual, J.; Flatken, M.; Félix, R.; Li, G.; Turren-Cruz, S. H.; Aldamasy, M. H.; Hartmann, C.; Li, M.; Di Girolamo, D.; Nasti, G.; Hüsam, E.; Wilks, R. G.; Dallmann, A.; Bär, M.; Hoell, A.; Abate, A. Fluoride Chemistry in Tin Halide Perovskites. *Angew. Chem., Int. Ed.* **2021**, *60* (39), 21583–21591. https://doi.org/10.1002/anie.202107599.

(5) Yuan, F.; Zheng, X.; Johnston, A.; Wang, Y.-K.; Zhou, C.; Dong, Y.; Chen, B.; Chen, H.; Fan, J. Z.; Sharma, G.; Li, P.; Gao, Y.; Voznyy, O.; Kung, H.-T.; Lu, Z.-H.; Bakr, O. M.; Sargent, E. H.

Color-Pure Red Light-Emitting Diodes Based on Two-Dimensional Lead-Free Perovskites. *Sci. Adv.* **2020**, *6*, 253–267. https://doi.org/10.1126/sciadv.abb0253.

(6) Triggs, C. T.; Ross, R. D.; Mihalyi-Koch, W.; Clewett, C. F. M.; Sanders, K. M.; Guzei, I. A.; Jin, S. Spacer Cation Design Motifs for Enhanced Air Stability in Lead-Free 2D Tin Halide Perovskites. *ACS Energy Lett* **2024**, 1835–1843. https://doi.org/10.1021/acsenergylett.4c00615.

(7) Sheldrick, G. M. SHELXT - Integrated Space-Group and Crystal-Structure Determination. *Acta Crystallogr. A* **2015**, *71* (1), 3–8. https://doi.org/10.1107/S2053273314026370.

(8) Sheldrick, G. M. Crystal Structure Refinement with SHELXL. *Acta Crystallogr. C Struct. Chem.* **2015**, *71*, 3–8. https://doi.org/10.1107/S2053229614024218.

(9) Dolomanov, O. V.; Bourhis, L. J.; Gildea, R. J.; Howard, J. A. K.; Puschmann, H. OLEX2: A Complete Structure Solution, Refinement and Analysis Program. *J Appl. Crystallogr.* **2009**, *42* (2), 339–341. https://doi.org/10.1107/S0021889808042726.

(10) Momma, K.; Izumi, F. VESTA: A Three-Dimensional Visualization System for Electronic and Structural Analysis. *J Appl Crystallogr* **2008**, *41* (3), 653–658. https://doi.org/10.1107/S0021889808012016.

(11) Li, Y.; Zhou, H.; Xia, M.; Shen, H.; Wang, T.; Gao, H.; Sheng, X.; Han, Y.; Chen, Z.; Dou, L.; Zhu, H.; Shi, E. Phase-Pure 2D Tin Halide Perovskite Thin Flakes for Stable Lasing. *Sci. Adv.* **2023**, *9*. https://doi.org/10.1126/sciadv.adh0517.

(12) Li, X.; Guan, Y.; Li, X.; Fu, Y. Stereochemically Active Lone Pairs and Nonlinear Optical Properties of Two-Dimensional Multilayered Tin and Germanium Iodide Perovskites. *J. Am. Chem. Soc.* **2022**, *144* (39), 18030-18043. https://doi.org/10.1021/jacs.2c07535.

(13) Sun, M.; Ma, M.; Guo, Y.; Yuan, S.; Xiong, H.; Tan, Z.; Li, W.; Fan, J.; Ning, Z. Difluorine-Substituted Molecule-Based Low-Dimensional Structure for Highly Stable Tin Perovskite Solar Cells. *Solar RRL* **2022**, *6*, 2200672. https://doi.org/10.1002/solr.202200672.

(14) Li, X.; Fu, Y.; Pedesseau, L.; Guo, P.; Cuthriell, S.; Hadar, I.; Even, J.; Katan, C.; Stoumpos, C. C.; Schaller, R. D.; Harel, E.; Kanatzidis, M. G. Negative Pressure Engineering with Large Cage Cations in 2D Halide Perovskites Causes Lattice Softening. *J. Am. Chem. Soc.* **2020**, *142* (26), 11486–11496. https://doi.org/10.1021/jacs.0c03860.

(15) Spanopoulos, I.; Hadar, I.; Ke, W.; Tu, Q.; Chen, M.; Tsai, H.; He, Y.; Shekhawat, G.; Dravid, V. P.; Wasielewski, M. R.; Mohite, A. D.; Stoumpos, C. C.; Kanatzidis, M. G. Uniaxial Expansion of the 2D Ruddlesden-Popper Perovskite Family for Improved Environmental Stability. *J. Am. Chem. Soc.* **2019**, *141* (13), 5518–5534. https://doi.org/10.1021/jacs.9b01327.

(16) Han, S.; Yao, Y.; Liu, X.; Li, B.; Ji, C.; Sun, Z.; Hong, M.; Luo, J. Highly Oriented Thin Films of 2D Ruddlesden-Popper Hybrid Perovskite toward Superfast Response Photodetectors. *Small* **2019**, *15* (39), 1901194. https://doi.org/10.1002/smll.201901194.

(17) Yang, T.; Li, Y.; Han, S.; Liu, Y.; Xu, Z.; Li, M.; Wang, J.; Ma, Y.; Luo, J.; Sun, Z. Exploiting Two-Dimensional Hybrid Perovskites Incorporating Secondary Amines for High-Performance Array Photodetection. *J Mater. Chem. C Mater.* **2020**, *8* (37), 12848–12853. https://doi.org/10.1039/d0tc03382k.

(18) Mihalyi-Koch, W.; Guo, S.; Dai, Z.; Pan, D.; Lafayette, D. P.; Scheeler, J. M.; Sanders, K. M.; Teat, S. J.; Wright, J. C.; Lü, X.; Rappe, A. M.; Jin, S. Revealing Hidden Non-Centrosymmetry in Globally Centrosymmetric 2D Halide Perovskites. *Chem* **2024**, *10* (7), 2180-2195. https://doi.org/10.1016/j.chempr.2024.03.012.

(19) Xu, Z.; Li, Y.; Liu, X.; Ji, C.; Chen, H.; Li, L.; Han, S.; Hong, M.; Luo, J.; Sun, Z. Highly Sensitive and Ultrafast Responding Array Photodetector Based on a Newly Tailored 2D Lead Iodide Perovskite Crystal. *Adv. Opt. Mater.* **2019**, *7* (11), 1900308. https://doi.org/10.1002/adom.201900308.

(20) Yan, J.; Zhang, W.; Geng, S.; Qiu, C.; Chu, Y.; Meng, R.; Zeng, P.; Liu, M.; Xiao, Z.; Hu, Y. Electronic State Modulation by Large A-Site Cations in Quasi-Two-Dimensional Organic-Inorganic Lead Halide Perovskites. *Chem. Mater.* **2023**, *35* (1), 289–294. https://doi.org/10.1021/acs.chemmater.2c03189.

(21) Wang, Y.; Liu, X.; Li, L.; Ji, C.; Sun, Z.; Han, S.; Tao, K.; Luo, J. (C 6 H 13 NH 3) 2 (NH 2 CHNH 2)Pb 2 I 7 : A Two-Dimensional Bilayer Inorganic–Organic Hybrid Perovskite Showing Photodetecting Behavior. *Chem. Asian J.* **2019**, *14* (9), 1530–1534. https://doi.org/10.1002/asia.201900059.

(22) Fu, Y.; Hautzinger, M. P.; Luo, Z.; Wang, F.; Pan, D.; Aristov, M. M.; Guzei, I. A.; Pan, A.; Zhu, X.; Jin, S. Incorporating Large A Cations into Lead Iodide Perovskite Cages: Relaxed Goldschmidt Tolerance Factor and Impact on Exciton-Phonon Interaction. *ACS Cent. Sci.* **2019**, *5* (8), 1377–1386. https://doi.org/10.1021/acscentsci.9b00367.

(23) Vasileiadou, E. S.; Hadar, I.; Kepenekian, M.; Even, J.; Tu, Q.; Malliakas, C. D.; Friedrich, D.; Spanopoulos, I.; Hoffman, J. M.; Dravid, V. P.; Kanatzidis, M. G. Shedding Light on the Stability and Structure-Property Relationships of Two-Dimensional Hybrid Lead Bromide Perovskites. *Chem. Mater.* **2021**, *33* (13), 5085–5107. https://doi.org/10.1021/acs.chemmater.1c01129.

(24) Skorokhod, A.; Quarti, C.; Abhervé, A.; Allain, M.; Even, J.; Katan, C.; Mercier, N. Halide Containing Short Organic Monocations in n = 1-4 2D Multilayered Halide Perovskite Thin Films and Crystals. *Chem. Mater.* **2023**, *35* (7), 2873–2883. https://doi.org/10.1021/acs.chemmater.2c03718.

(25) Fu, D.; Zhang, Y.; Chen, Z.; Pan, L.; He, Y.; Luo, J. Bulk Photovoltaic Effect Induced by Non-Covalent Interactions in Bilayered Hybrid Perovskite for Efficient Passive X-Ray Detection. *Small* **2024** *20*, 2403198. https://doi.org/10.1002/smll.202403198.

(26) Zhuang, J. chang; Wei, W. juan; Song, N.; Tang, Y. zhi; Tan, Y. hui; Han, D. chong; Li, Y. kong. A Narrow Bandgap of 2D Ruddlesden-Popper Bilayer Perovskite with Giant Entropy Change and Photoluminescence. *Chem. – Eur. J.* **2021**, *27* (63), 15716–15721. https://doi.org/10.1002/chem.202102550.

(27) Gilley, I. W.; Kwon, H. W.; Liu, C.; Yang, Y.; Huang, C.; Wan, H.; Bati, A. S. R.; Oriel, E. H.; Kepenekian, M.; Vishal, B.; Zeiske, S.; Bayikadi, K. S.; Wiggins, T. E.; Vasileiadou, E. S.; Chen, B.; Schaller, R. D.; Even, J.; De Wolf, S.; Sargent, E. H.; Kanatzidis, M. G. Combining Organic Cations of Different Sizes Grants Improved Control over Perovskitoid Dimensionality and Bandgap. *J. Am. Chem. Soc.* **2025**, *147* (9), 7777-7787. https://doi.org/10.1021/jacs.4c17654.

(28) Oswald, I. W. H.; Koegel, A. A.; Neilson, J. R. General Synthesis Principles for Ruddlesden-Popper Hybrid Perovskite Halides from a Dynamic Equilibrium. *Chem. Mater.* **2018**, *30* (23), 8606–8614. https://doi.org/10.1021/acs.chemmater.8b03817.

(29) Hao, Y.; Qiu, Z.; Zhang, X.; Wei, Z.; Yao, J.; Cai, H. Series of 2D Multilayered Perovskites Constructed by Slicing the 3D [(CH3NH3)PbI3] with 4-Fluorobenzylamine. *Inorg. Chem. Commun.* **2018**, *97*, 134–138. https://doi.org/10.1016/j.inoche.2018.09.030.

(30) Chen, Y.; Tang, L.; Liu, Y.; Yang, T.; Hua, L.; Zeng, X.; Luo, J.; Sun, Z. Polar 2D Hybrid Perovskite Crystals with Intrinsic Strong Linear Dichroism for Polarization-Sensitive and Self-Powered Detection. *J. Mater. Chem. C Mater.* **2023**, *11* (12), 3981–3988. https://doi.org/10.1039/d3tc00199g.

(31) Stoumpos, C. C.; Mao, L.; Malliakas, C. D.; Kanatzidis, M. G. Structure-Band Gap Relationships in Hexagonal Polytypes and Low-Dimensional Structures of Hybrid Tin Iodide Perovskites. *Inorg. Chem*. **2017**, *56*, 1, 56-73. https://doi.org/10.1021/acs.inorgchem.6b02764.

(32) Mao, L.; Morgan, E. E.; Li, A.; Kennard, R. M.; Hong, M. J.; Liu, Y.; Dahlman, C. J.; Labram, J. G.; Chabinyc, M. L.; Seshadri, R. Layered Hybrid Lead Iodide Perovskites with Short Interlayer Distances. *ACS Energy Lett.* **2022**, *7* (8), 2801–2806. https://doi.org/10.1021/acsenergylett.2c01321.

(33) Pan, X.; Chen, H.; Lu, L.; Han, S.; Ma, Y.; Wang, J.; Guo, W.; Xu, H.; Luo, J.; Sun, Z. Incorporating Guanidinium as Perovskitizer-Cation of Two-Dimensional Metal Halide for Crystal-Array Photodetectors. *Chem. Asian J.* **2021**, *16*, 1925–1929. https://doi.org/10.1002/asia.202100425.

(34) Zhang, J.; Guo, W.; Xu, H.; Fan, Q.; Wei, L.; Zhao, X.; Sun, Z.; Luo, J. A Polar Multilayered Two-Dimensional Hybrid Perovskite for Self-Driven X-Ray Photodetection with a Low Detection Limit. *Mater. Chem. Front.* **2024**, *8*, 3807-3816. https://doi.org/10.1039/d4qm00582a.

(35) Huang, P. J.; Taniguchi, K.; Miyasaka, H. Bulk Photovoltaic Effect in a Pair of Chiral-Polar Layered Perovskite-Type Lead Iodides Altered by Chirality of Organic Cations. *J. Am. Chem. Soc.* **2019**, *141* (37), 14520–14523. https://doi.org/10.1021/jacs.9b06815.

(36) Xia, M.; Wang, T.; Lu, Y.; Li, Y.; Li, B.; Shen, H.; Guo, Y.; Yu, Y.; Dong, J.; Dou, L.; Liu, Y.; Shi, E. Kinetic Wulff-Shaped Heteroepitaxy of Phase-Pure 2D Perovskite Heterostructures with Deterministic Slab Thickness. *Nat. Synth.* **2025**, *4*, 380-390. https://doi.org/10.1038/s44160-024-00692-5.

(37) Park, I. H.; Chu, L.; Leng, K.; Choy, Y. F.; Liu, W.; Abdelwahab, I.; Zhu, Z.; Ma, Z.; Chen, W.; Xu, Q. H.; Eda, G.; Loh, K. P. Highly Stable Two-Dimensional Tin(II) Iodide Hybrid Organic–Inorganic Perovskite Based on Stilbene Derivative. *Adv. Funct. Mater.* **2019**, *29* (39), 1904810. https://doi.org/10.1002/adfm.201904810.

(38) Papavassiliou, G. C.; Mousdis, G. A.; Raptopoulou, C. P.; Terzis, A. Some New Luminescent Compounds Based on 4-Methylbenzylamine and Lead Halides. *Z. Naturforsch. B.* **2000**, *55* (6), 536–540. https://doi.org/doi:10.1515/znb-2000-0615.

(39) Morrow, D. J.; Hautzinger, M. P.; Lafayette, D. P.; Scheeler, J. M.; Dang, L.; Leng, M.; Kohler, D. D.; Wheaton, A. M.; Fu, Y.; Guzei, I. A.; Tang, J.; Jin, S.; Wright, J. C. Disentangling Second Harmonic Generation from Multiphoton Photoluminescence in Halide Perovskites Using

Multidimensional Harmonic Generation. *J. Phys. Chem. Lett.* **2020**, *11* (16), 6551–6559. https://doi.org/10.1021/acs.jpclett.0c01720.

(40) Slavney, A. H.; Smaha, R. W.; Smith, I. C.; Jaffe, A.; Umeyama, D.; Karunadasa, H. I. Chemical Approaches to Addressing the Instability and Toxicity of Lead-Halide Perovskite Absorbers. *Inorg. Chem.* **2017**, *56* (1), 46–55. https://doi.org/10.1021/acs.inorgchem.6b01336.

(41) Venkatesan, N. R.; Mahdi, A.; Barraza, B.; Wu, G.; Chabinyc, M. L.; Seshadri, R. Enhanced Yield-Mobility Products in Hybrid Halide Ruddlesden-Popper Compounds with Aromatic Ammonium Spacers. *Dalton Trans.* **2019**, *48* (37), 14019–14026. https://doi.org/10.1039/c9dt03074c.

(42) Park, J. Y.; Song, R.; Liang, J.; Jin, L.; Wang, K.; Li, S.; Shi, E.; Gao, Y.; Zeller, M.; Teat, S. J.; Guo, P.; Huang, L.; Zhao, Y. S.; Blum, V.; Dou, L. Thickness Control of Organic Semiconductor-Incorporated Perovskites. *Nat. Chem.* **2023**, *15*, 1745-1753. https://doi.org/10.1038/s41557-023-01311-0.

(43) Mercier, N. (HO2C(CH2)3NH3)2(CH3NH3)Pb2I7: A Predicted Noncentrosymmetrical Structure Built up from Carboxylic Acid Supramolecular Synthons and Bilayer Perovskite Sheets. *CrystEngComm* **2005**, *7*, 429–432. https://doi.org/10.1039/b504342e.

(44) Pariari, D.; Pramanik, T.; Rohj, R. K.; Vidhan, A.; Sarkar, S. K.; Sarma, D. D. New Homologous Series of the 2D Hybrid Lead Iodide System and Its Implications for Photovoltaic Applications. *Energy Fuels* **2025**, *39* (14), 7021-7030. https://doi.org/10.1021/acs.energyfuels.4c05881.

(45) ZhenTao, J.; Yanhuan, H.; Hui, W.; Xiuxiu, Z.; Jiaojiao, Y.; Mingli, L.; Zhenhong, W. Two Bilayer Organic-Inorganic Hybrid Perovskite Compounds Exhibiting Reversible Phase Transition and Dielectric Anomalies. *J. Solid State Chem.* **2020**, *282*, 121104. https://doi.org/10.1016/j.jssc.2019.121104.

(46) Vasileiadou, E. S.; Jiang, X.; Kepenekian, M.; Even, J.; De Siena, M. C.; Klepov, V. V.; Friedrich, D.; Spanopoulos, I.; Tu, Q.; Tajuddin, I. S.; Weiss, E. A.; Kanatzidis, M. G. Thick-Layer Lead Iodide Perovskites with Bifunctional Organic Spacers Allylammonium and Iodopropylammonium Exhibiting Trap-State Emission. *J. Am. Chem. Soc.* **2022**, *144* (14), 6390–6409. https://doi.org/10.1021/jacs.2c00571.

(47) Lai, H.; Lu, D.; Xu, Z.; Zheng, N.; Xie, Z.; Liu, Y. Organic-Salt-Assisted Crystal Growth and Orientation of Quasi-2D Ruddlesden–Popper Perovskites for Solar Cells with Efficiency over 19%. *Adv. Mater.* **2020**, *32* (33), 2001470. https://doi.org/10.1002/adma.202001470.

(48) You, S.; Yu, P.; Wu, J.; Zhu, Z. K.; Guan, Q.; Li, L.; Ji, C.; Liu, X.; Luo, J. Weak X-Ray to Visible Lights Detection Enabled by a 2D Multilayered Lead Iodide Perovskite with Iodine-Substituted Spacer. *Adv. Sci.* **2023**, *10* (21), 2301149. https://doi.org/10.1002/advs.202301149.

(49) Mihalyi-Koch, W.; Dai, Z.; Sun, M.-J.; Park, J. Y.; Lafayette, D.; Sanders, K.; Guzei, I.; Wright, J.; Huang, L.; Rappe, A.; Jin, S. Multifunctional Polar 2D Lead Iodide Perovskites Exhibiting Persistent Spin Texture. *J. Am. Chem. Soc.* **2025**, *147*, 26, 23079-23089. https://doi.org/10.1021/jacs.5c06227.

(50) Mihalyi-Koch, W.; Folpini, G.; Roy, C. R.; Kaiser, W.; Wu, C.-S.; Sanders, K. M.; Guzei, I. A.; Wright, J. C.; De Angelis, F.; Cortecchia, D.; Petrozza, A.; Jin, S. Tuning Structure and Excitonic Properties of 2D Ruddlesden–Popper Germanium, Tin, and Lead Iodide Perovskites via Interplay between Cations. *J. Am. Chem. Soc.* **2023**, *145* (51), 28111-28123. https://doi.org/10.1021/jacs.3c09793.

(51) Guo, S.; Mihalyi-Koch, W.; Mao, Y.; Li, X.; Bu, K.; Hong, H.; Hautzinger, M. P.; Luo, H.; Wang, D.; Gu, J.; Zhang, Y.; Zhang, D.; Hu, Q.; Ding, Y.; Yang, W.; Fu, Y.; Jin, S.; Lü, X. Exciton Engineering of 2D Ruddlesden–Popper Perovskites by Synergistically Tuning the Intra and Interlayer Structures. *Nat. Commun.* **2024**, *15* (1), 3001. https://doi.org/10.1038/s41467-024-47225-4.

(52) Ye, H.; Peng, Y.; Wei, M.; Zhang, X.; Zhu, T.; Guan, Q.; Li, L.; Chen, S.; Liu, X.; Luo, J. Bulk Photovoltaic Effect in Chiral Layered Hybrid Perovskite Enables Highly Sensitive Near-Infrared Circular Polarization Photodetection. *Chem. Mater.* **2023**, *35* (17), 6591–6597. https://doi.org/10.1021/acs.chemmater.2c03770.
